# PolyProline Predictor: A web server for empirical sequence‐based prediction of polyproline II helices

**DOI:** 10.1002/pro.70675

**Published:** 2026-06-07

**Authors:** Rubén López‐Sánchez, David Pantoja‐Uceda, Miguel Mompeán, Douglas V. Laurents

**Affiliations:** ^1^ Departamento de Química Física Biológica Instituto de Química Física “Blas Cabrera”—CSIC Madrid Spain

**Keywords:** CD spectroscopy, molecular dynamics, mycobacteria PE_PGRS, NMR spectroscopy, polyproline II helix, RIPK3, webserver

## Abstract

Polyproline II (PPII) helices are extended left‐handed secondary structures increasingly recognized for their roles in molecular recognition, signaling and within intrinsically disordered regions of proteins. Despite their functional importance, predicting regions with propensity to form PPII helices from sequence alone remains challenging due to subtle sequence determinants and their frequent misclassification as random coil. Here, we present PolyProline Predictor (PPP), a user‐friendly web server (https://rmni.iqf.csic.es/software/polypropre/) for empirical, sequence‐based prediction of PPII helices. Unlike machine learning approaches, PPP aligns query sequences against a curated database of experimentally validated PPII helices, providing an interpretable, composition‐, and position‐sensitive similarity map. PPP successfully identified conserved PPII motifs in diverse proteins, and predicted the presence of similar motifs in regions lacking experimental structures but modeled by AlphaFold as extended PPII conformations, such as glycine‐rich plant proteins, mycobacterial PE_PGRS virulence factors, and the “disordered” C‐terminal tails of GroEL and its homologs, as well as the amyloid‐flanking region of the necroptosis effector RIPK3. Molecular dynamics simulations further supported persistent PPII helical bundles in three glycine‐rich mycobacterial proteins and more heterogeneous, transient PPII populations in plant proteins and RIPK3. Circular dichroism and nuclear magnetic resonance (NMR) spectroscopy validated these predictions for RIPK3, revealing partially populated PPII conformations flanking its amyloid core. Such motifs may regulate its amyloid assembly, offering structural insight into mechanisms of functional amyloid formation. By combining experimental evidence with interpretable prediction, PPP fills a critical gap in bioinformatics tools and enables systematic exploration of regions with propensity to form PPII helices across proteomes, redefining the structural landscape of low‐complexity regions.

## 1 INTRODUCTION

Polyproline II (PPII) helices are extended, left‐handed structures increasingly recognized for their versatile roles in protein architecture and function (Adzhubei et al., 2013). Beyond proline‐rich sequences, many glycine‐rich regions also adopt the PPII conformation, contributing to structural diversity in both folded and intrinsically disordered proteins (IDPs) or regions (IDRs) (Rodríguez & Laurents, 2024).

PPII helices are implicated in diverse biological processes. This conformation is preferred in conformational ensembles rich in proline (Tiffany & Krimm, 1968a), Lys, Glu (Tiffany & Krimm, 1968b), Gly (Crick & Rich, 1955), Met or Ala (Hagarman et al., 2010) residues. They can drive phase separation and the formation of bimolecular condensates by mediating interactions with other PPII segments helices or folded modules like SH3 or WW domains (Li, Banjade, et al., 2012; Mompeán, Oroz, & Laurents, 2021). Recent studies have shown that glycine‐rich sequences in proteins such as FUS can form PPII helices that might assemble to contribute to stabilizing condensates (Kar et al., 2021; Mompeán, McAvan, et al., 2021). The structural versatility of PPII helices and the increasing number of PPII helical bundle domains being discovered in nature (Rodríguez & Laurents, 2024) suggests they could serve as an alternative structural element for protein design (Seco et al., 2026). This could be facilitated by leveraging advanced machine learning tools which have recently shown success with enzymes composed of α‐helices and β‐strands (Sumida et al., 2024). For instance, the snow flea antifreeze protein (sfAFP) (RCSB PDB ID 3BOG) (Pentelute et al., 2008) assembles into stable glycine‐rich PPII helical bundles stabilized by canonical and non‐canonical hydrogen bonds as well as dimerization (Treviño et al., 2018), opening new avenues for engineering functional higher‐order protein structures.

Despite their biological and biophysical relevance, predicting PPII helices from amino acid sequences remains challenging. Unlike α‐helices and β‐sheets, PPII helices encompass structurally diverse states, ranging from isolated and often weakly populated conformations in many proline‐rich segments to substantially populated, persistent assemblies in glycine‐rich PPII helical bundles. This heterogeneity makes them difficult to capture with conventional sequence‐based predictors, which often subsume them into generic coil or disorder categories rather than recognizing them as PPII‐prone segments. The report from computational studies supported by nuclear magnetic resonance (NMR) chemical shifts of significant stabilization from hydrogen bond cooperativity (López‐Sánchez et al., 2024; López‐Sánchez et al., 2025) implies that glycine‐rich PPII helical bundles could be more common than is currently suggested by their relative scarcity in the RCSB PDB, which is dominated by rigid or crystallizable proteins amenable for study by CryoEM or x‐ray diffraction. Early resources like PolyprOnline (Chebrek et al., 2014) compiled PPII assignments from solved protein structures and offered interactive querying and visualization, integrating several assignment programs (XTLSSTR, PROSS, SEGNO) along with a DSSP‐based PPII method. More recent tools include PPIIPRED (O'Brien et al., 2020), a bidirectional recurrent neural network predictor available on‐line, while deep‐learning approaches (BERT‐PPII and AAindex‐PPII) achieve higher accuracy but lack accessible web implementation (Feng et al., 2022; He et al., 2023). General‐purpose secondary‐structure on‐line tools typically output 3‐state (Q3) or 8‐state (Q8) labels and do not include PPII as a native class (Høie et al., 2022; Singh et al., 2022), with notable exceptions such as the DynaMine predictor (Kagami et al., 2021), which employs regression models, or the DSSP 4 structure‐based assigner (Hekkelman et al., 2025). This leaves a clear gap for an interpretable, alignment‐based web server that leverages experimentally validated PPII structural data to enable accurate, sequence‐based prediction. In particular, there is a need for tools that can capture local sequence signatures of PPII propensity in glycine‐rich or low‐complexity regions, as conventional predictors often perform better on canonical proline‐rich PPII segments and may otherwise classify these regions as disordered or ambiguously assign them to conventional secondary‐structure categories.

To address this need, we developed *PolyProline Predictor (PPP)*, a publicly accessible web server (https://rmni.iqf.csic.es/software/polypropre/) that evaluates the likelihood of PPII helix formation from amino acid sequences. PPP analyzes experimentally determined protein structures in the RCSB PDB to identify residues with dihedral angles matching known PPII helices. By aligning user‐submitted sequences with these validated segments, PPP calculates a profile of similarity to experimentally validated PPII segments. This approach directly incorporates experimental structural data, enhancing reliability and detecting subtle, sequence‐specific tendencies that generalized neural network approaches might miss. Thus, PPP is designed not only to predict putative PPII helices and bundles, but also to provide an interpretable framework for prioritizing candidate segments for subsequent validation.

Using PPP, we have detected numerous sequences in biologically relevant proteins which putatively adopt PPII helices. Among them, we focused the receptor‐interacting protein kinase 3 (RIPK3) (Uniprot ID Q9Y572) because its intrinsically disordered C‐terminus contains the RIP homotypic interaction motif (RHIM), which forms physiological amyloid fibrils critical for programmed cell death (Wu et al., 2021). The region immediately preceding the RHIM, known as the pre‐RHIM, acts as a regulatory segment for assembly and is considered structurally disordered (Pham et al., 2023). This makes RIPK3 an interesting test case for PPP, as any local PPII propensity detected within this region would not be obvious from the global structural description alone yet could have functional implications for amyloid assembly.

Interestingly, PPP predicted two discrete PPII‐prone segments within this pre‐RHIM region, challenging the assumption of complete disorder. Given that transient PPII structures can influence protein–protein interactions and assembly pathways (Bhattacharyya et al., 2006) these elements could modulate RHIM amyloid formation. Here, we computationally and experimentally validate PPP's prediction using molecular dynamics (MD) simulations together with circular dichroism (CD) and NMR spectroscopy, confirming that these motifs adopt partially populated PPII conformations in solution. More broadly, this combined strategy allows us to assess whether PPP can reveal structurally meaningful PPII tendencies in biologically relevant systems that would otherwise remain cryptic in static models or conventional disorder annotations. Our results underscore the potential of PPP to reveal hidden structural propensities in disordered regions, with implications for understanding and predicting functional protein assemblies.

## 2 RESULTS

### 2.1 Global analysis of PPII helices in the RCSB PDB

**FIGURE 1:**
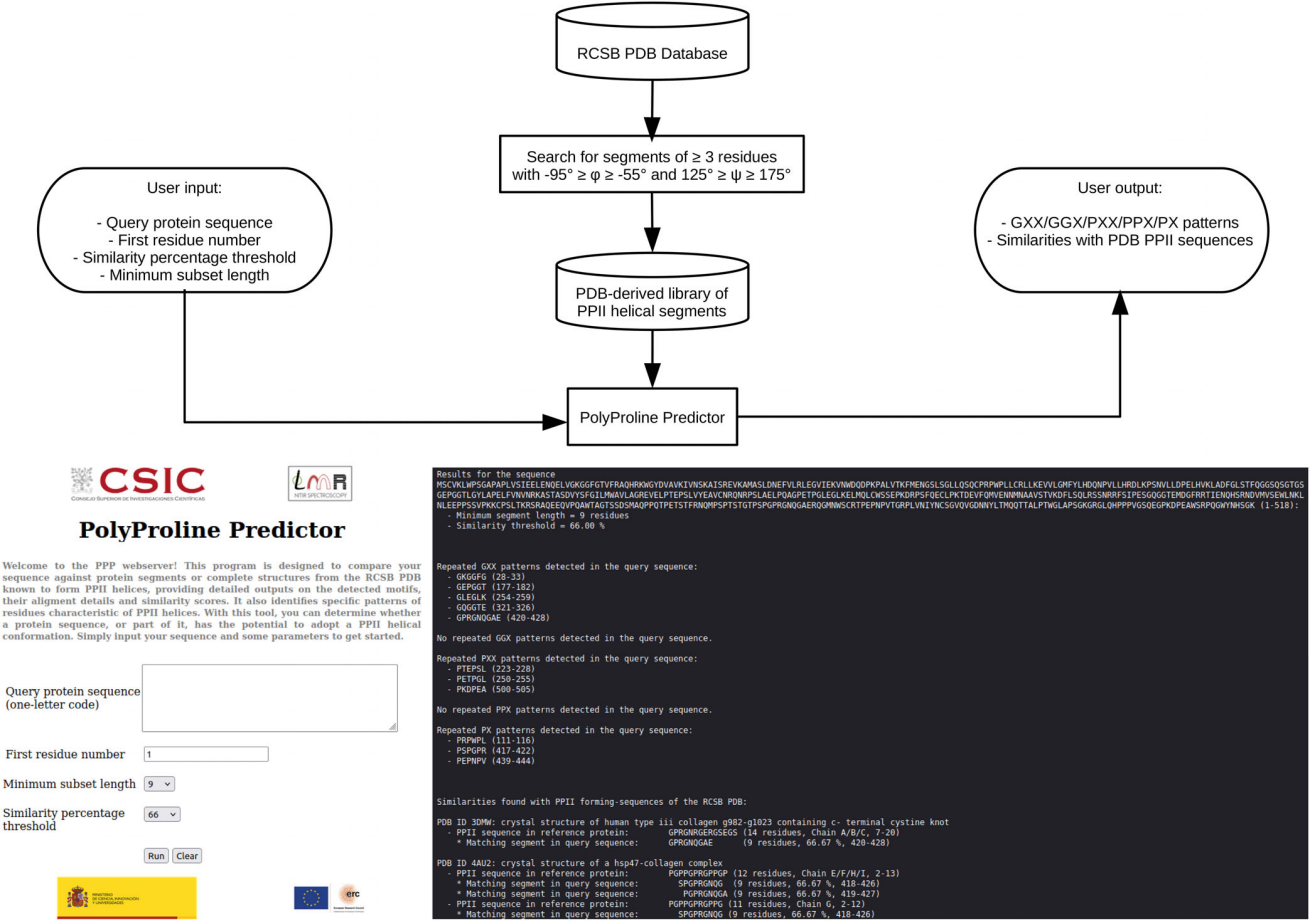
PolyProline Predictor engine. (Top) Process flow diagram of PPP, emphasizing the development of a library of RCSB PDB‐derived PPII helical segments, which is searched to obtain PPII structural segments whose sequences are highly similar to query sequence. (Bottom) PPP user interface for input at the left and output at the right for the RIPK3 example seen afterwards. The output of this figure is only showing some similarities to the PPII helix‐forming sequences in the RCSB PDB, not all of them.

To assess the distribution of PPII helices in known structures, we analyzed the entire RCSB PDB. The study revealed that over 130,000 entries contain at least one PPII helical turn (three residues), more than 4000 with at least two consecutive turns (six residues); 238 with at least three (nine residues) and only 22 entries exhibit segments with at least four (12 residues), reflecting the rarity of long PPII helices. This curated dataset serves as the foundation for rapid sequence alignment and prediction through the PPP web interface. The program's workflow and example web server input and output are shown in Figure 1. Further technical details are given in the Materials and Methods section.

### 2.2 PPP uncovers biologically relevant regions with propensity to form PPII helices

While testing our web server, we detected PPII helical bundles in the cyanophage tail spike fiber (RCSB PDB 7YFW) (Yang et al., 2023), in the tail wing brush domains (gp33), and in subdomains of the tail spike domains (gp29) of the mycobacteriophage Bxb1 (RCSB PDB ID 9D93) (Freeman et al., 2025). In the latter, gp33 was described as being “unique in both sequence and structure”. We note that these domains actually resemble the PPII helical bundle domain of the tail spike protein of *Salmonella* phage S16 (RCSB PDB 6F45) (Dunne et al., 2018).

**FIGURE 2:**
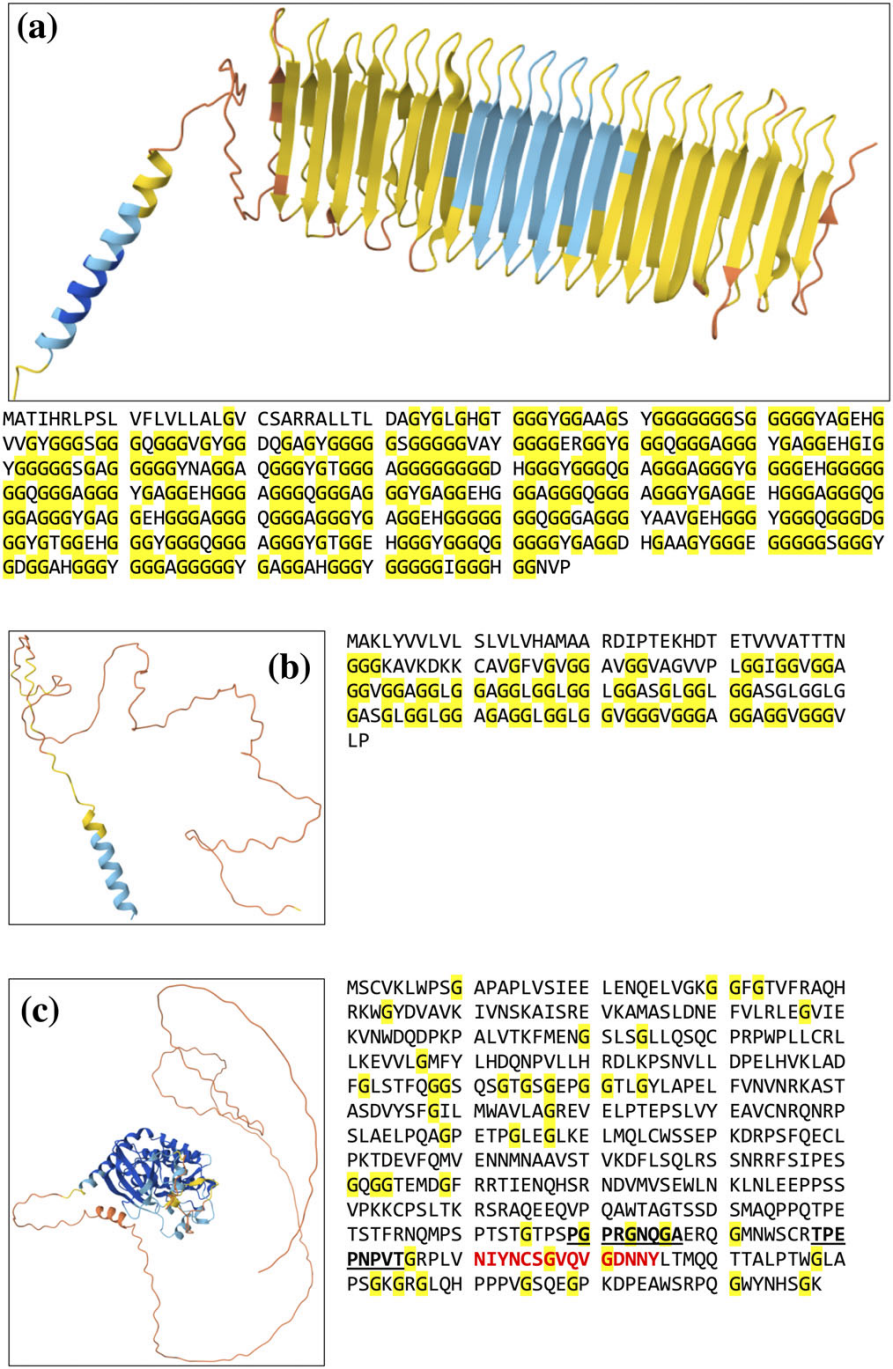
AlphaFold protein structural database models of plant glycine‐rich proteins. (a) Kidney bean cell wall protein (AF‐P10496‐F1‐v6), (b) Wild carrot embryo protein (AF‐Q39691‐F1‐v6), (c) Human RIPK3 (AlphaFold Server Model). Structural models are colored from blue, cyan, yellow, orange to red for very high to very low confidence according to the AlphaFold pIDDT score. Sequences are shown beneath the structural models with glycine residues highlighted in yellow. In panel C, the amyloid‐forming sequence is colored red and shown in bold and pre‐RHIM segments which form partly PPII helices (see below) are shown in bold and underlined.

Furthermore, we detected some notable cases of proteins with numerous GGX sequence motifs that match polyproline II helices in glycine‐rich PPII‐helical bundle domains, such as the Plant Class I Glycine‐Rich Proteins (Mangeon et al., 2010). These cases illustrate that PPP can reveal candidate PPII‐forming segments in biologically relevant glycine‐rich proteins even when their overall structural state remains uncertain or appears disordered in global structural models. One example is the glycine‐rich cell wall structural protein 1.8 from *Phaseolus vulgaris* (kidney bean; Uniprot ID P10496). The AlphaFold Protein Structure Database (AF PSD) (Jumper et al., 2021; Varadi et al., 2022) model for this protein shows with low confidence that it might adopt β‐sheets (Figure 2a). On the other hand, the glycine‐rich protein implicated in embryogenesis from *Daucus carota* (wild carrot; Uniprot ID Q39691) (Sato et al., 1995) is predicted to be disordered (Figure 2b). Likewise, plant class III and class IV glycine‐rich proteins, in particular *Arabidopsis thaliana* anthers protein (Uniprot ID Q9LY09) and *A. thaliana* cold shock response protein (Uniprot ID O65639), which have stretches of five to seven consecutive glycines, are also predicted to be disordered. Nevertheless, segments of several consecutive glycine residues have been reported to adopt PPII helices in the glycine‐rich PPII helical bundle domains in *Salmonella* bacteriophage S16 tail spike protein domain gp38 (RCSB PDB ID 6F45) (Dunne et al., 2018) and the human leukocyte tyrosine kinase or anaplastic lymphoma kinase (ALK) (RCSB PDB ID 7NX1 and 7N00) (De Munck et al., 2021; Reshetnyak et al., 2021). Remarkably, the AlphaFold Server (Abramson et al., 2024) predicts with moderate confidence that a hexaglycine peptide would bind and associate with the former. This suggests that glycine‐rich segments which are disordered in isolation might adopt a PPII helical conformation upon binding to glycine‐rich PPII helical bundles from other proteins (Figure S1). The presence of a large number of similar sequences suggests that many glycine‐rich regions with propensity to form PPII helices may be present in plants, underscoring the need for further corroboration.

Protein segments flanking amyloid cores have been shown to play decisive roles in the formation, energetics, and polymorphism of pathological amyloids, like Aβ in Alzheimer's disease (Jonsson et al., 2012), polyPro of Huntintin, Tau, and TDP‐43 (Bhopatkar & Kayed, 2023) but their possible roles in functional amyloids is much less understood. The RHIM motif (residues 448–472) in human RIPK3 forms amyloid fibrils essential for necroptotic signaling (Li, McQuade, et al., 2012; Mompeán et al., 2018) (Figure 2c). During previous NMR studies of this protein's C‐terminal disordered region, we discovered a cryptic region, the “pre‐RHIM,” involved in the assembly of RIPK3 with no significant tendency to adopt α‐helical or β‐sheet structure, but which showed enhanced rigidity, that was regarded a disordered segment (Pham et al., 2023), in agreement with the AF server model (Figure 2c). We subjected the entire C‐terminal segment (residues 387–518), encompassing the RHIM and its flanking regions, to the PPP web server. PPP remarkably identified two regions with high‐scoring propensity to form PPII helices flanking the RHIM within the pre‐RHIM domain: residues 419–427 (PGPRGNQGA) and 437–445 (RTPEPNPVT) (Figure 2c). These segments, rich in glycine and proline residues, border the β‐forming core (448–472) and suggest that PPP detects discrete, residue‐level PPII‐prone elements in a region that is globally classified as disordered, thereby providing structural insights that are not apparent from the AlphaFold model alone.

At this point, it is necessary to emphasize that PPP identified a high potential for PPII helix formation in the C‐termini of the bacterial chaperonin GroEL (Horovitz et al., 2022), which contains a highly conserved (GGM)_4_ motif and its mitochondrial (mHsp60) (Gomez‐Llorente et al., 2020) and chloroplastic (Cpn60) (Zhao et al., 2019) homologs. Although these segments are generally thought to be disordered, since they are not resolved in x‐ray crystallography or CryoEM studies (Gomez‐Llorente et al., 2020; Horovitz et al., 2022; Zhao et al., 2019; Zhao & Liu, 2018) their functional importance in substrate interaction suggests defined structural propensities (Weaver & Rye, 2014). Our recent structural characterization using MD simulations, in combination with CD and NMR spectroscopy, confirmed significant PPII helix populations in these segments, offering a novel structural hypothesis into their role in chaperonin function (Rodríguez et al., 2025). This previous validation is especially relevant here because it shows that PPP can successfully predict biologically meaningful PPII helices in regions that would otherwise be regarded as disordered tails. More broadly, the examples above illustrate that PPP can be used not only to detect putative PPII helices, but also to prioritize biologically relevant targets for computational and experimental validation.

**FIGURE 3:**
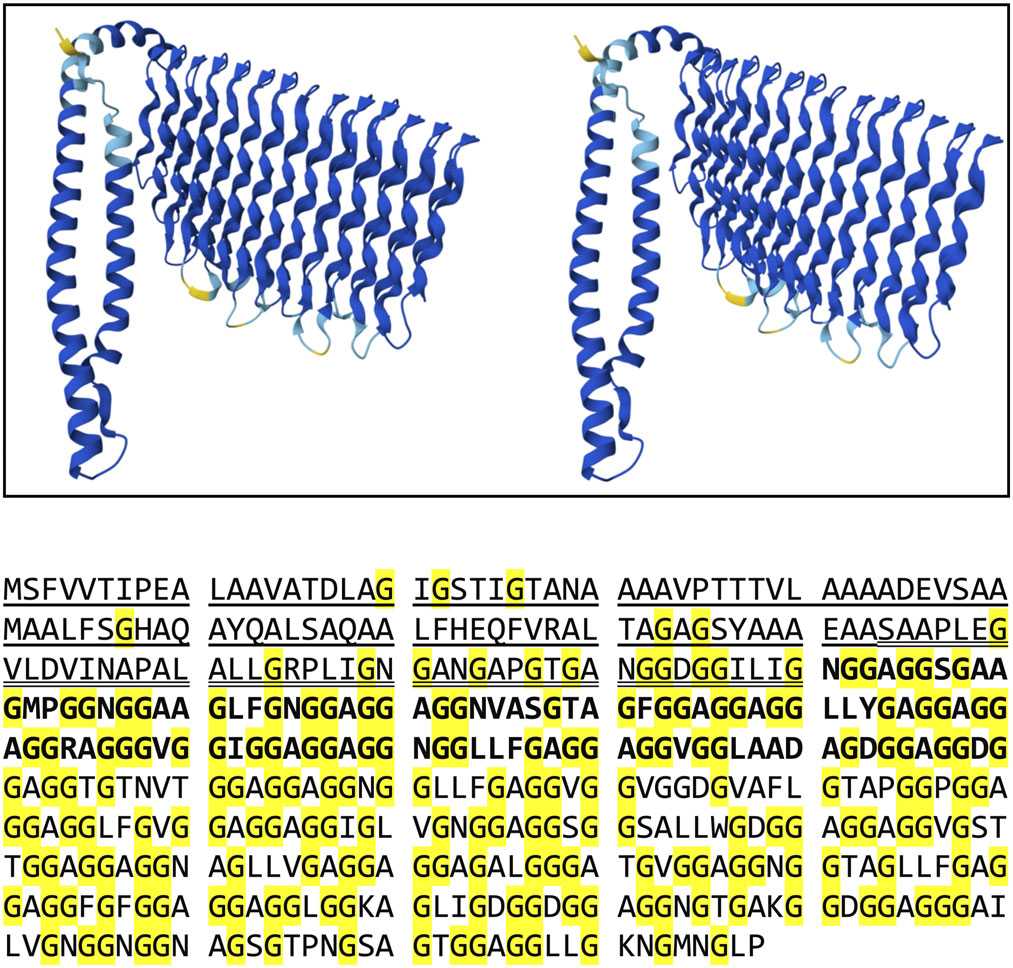
AlphaFold structural database model (crosseyed stereo view) of the mycobacterial protein PE_PGRS33 from *Mycobacterium tuberculosis* (AF‐P9WIF5‐F1‐v6). Blue, cyan, yellow, orange and red coloring indicates from very high to very low confidence according to the AlphaFold pIDDT score. Similar structural models are predicted by AlphaFold for PE_PGRS47 and Mmar_3581. In the sequence (below), the α‐helical PE domain sequence is underlined, the GRPLI linker sequence is double‐underlined, region of the PGRS domain reported to interact with TLR2 is shown in bold and glycine residues in the sequences are highlighted in yellow.

Pathological mycobacteria such as *M. tuberculosis* contain dozens of genes which encode PE_PGRS proteins and seem to play important roles in host immune system evasion and infection (Cole et al., 1998). The PPP server detects many of these sequences as matches of known PPII helical structures. This corroborates recent AF PSD models of PE_PGRS protein sequences, which show with high confidence that they form glycine‐rich PPII helical domains shaped like sails which have hydrophobic residues on one edge that protrude into the membrane (Berisio & Delogu, 2022). Thus, PPP independently identifies dense glycine‐rich regions with strong empirical similarity to experimentally observed PPII helices, providing a sequence‐based rationale for proposing PPII helical bundles in these proteins. These structural insights could shed light on the interaction of the glycine‐rich PPII helical bundle domain of *Mycobacterium tuberculosis* protein PE_PGRS33 (Uniprot ID P9WIF5) (Figure 3), with the host Toll Like Receptor 2, which is key for infection of macrophages (Palucci et al., 2016). Similarly, the PPP server also identifies large glycine‐rich segments in *M. tuberculosis* PE_PGRS47 (UniProt ID Q79FB3), implicated in host–pathogen interactions and intracellular persistence (Saini et al., 2016), and the corresponding AF PSD models further support the presence of a glycine‐rich PPII helical bundle domain in this protein. Furthermore, the PPP‐based detection of glycine‐rich sequences in *Mycobacterium marinum* protein Mmar_3581 (Uniprot ID B2HL21), which is key for f‐actin “rocket” based motility and is essential for cell to cell spread of infection (Hill & Welch, 2022), and the AF PSD model suggests that this protein also contains a glycine‐rich PPII helical bundle domain. Taken together, these examples indicate that PPP does not merely recover generic glycine‐richness, but pinpoints sequence patterns specifically associated with known PPII helices, whereas AF PSD provides a compatible structural framework at the global fold level.

### 2.3 Computational validation of PPII helices in plant, viral, bacterial, and human proteins

**FIGURE 4:**
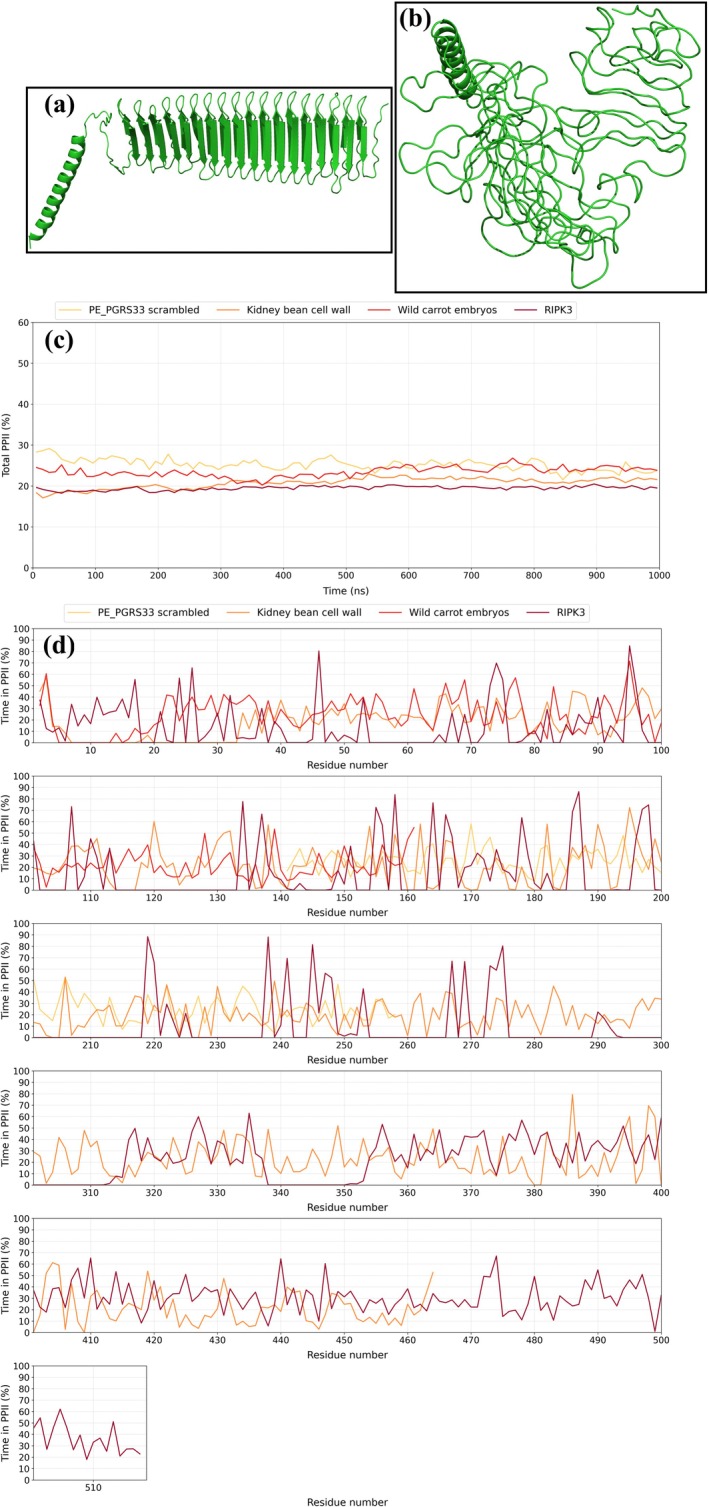
(a) First frame after equilibration (*t* = 0 ns); the AlphaFold Server model of the glycine rich protein from the bean *P. vulgaris*; featuring an N‐terminal α‐helix and a long β‐sheet. (b) Last frame of the simulation (*t* = 1000 ns) reveals structural retention for the α‐helix and loss of the β‐sheet structure. (c) Percent of PPII conformation for the scrambled TLR‐2 binding domain of *M. tuberculosis* PE_PGRS33 (residues 140–260) (yellow), the glycine‐rich proteins from *P. vulgaris* (orange) and the carrot *D. carota* (red) as well as human RIPK3 (reddish brown) during the simulation. (d) Per‐residue average PPII populations over the course of the simulations for *M. tuberculosis* PE_PGRS33 (residues 140–260) (yellow), the glycine‐rich proteins from *P. vulgaris* (orange) and the carrot *D. carota* (red) as well as human RIPK3 (reddish brown).

**FIGURE 5:**
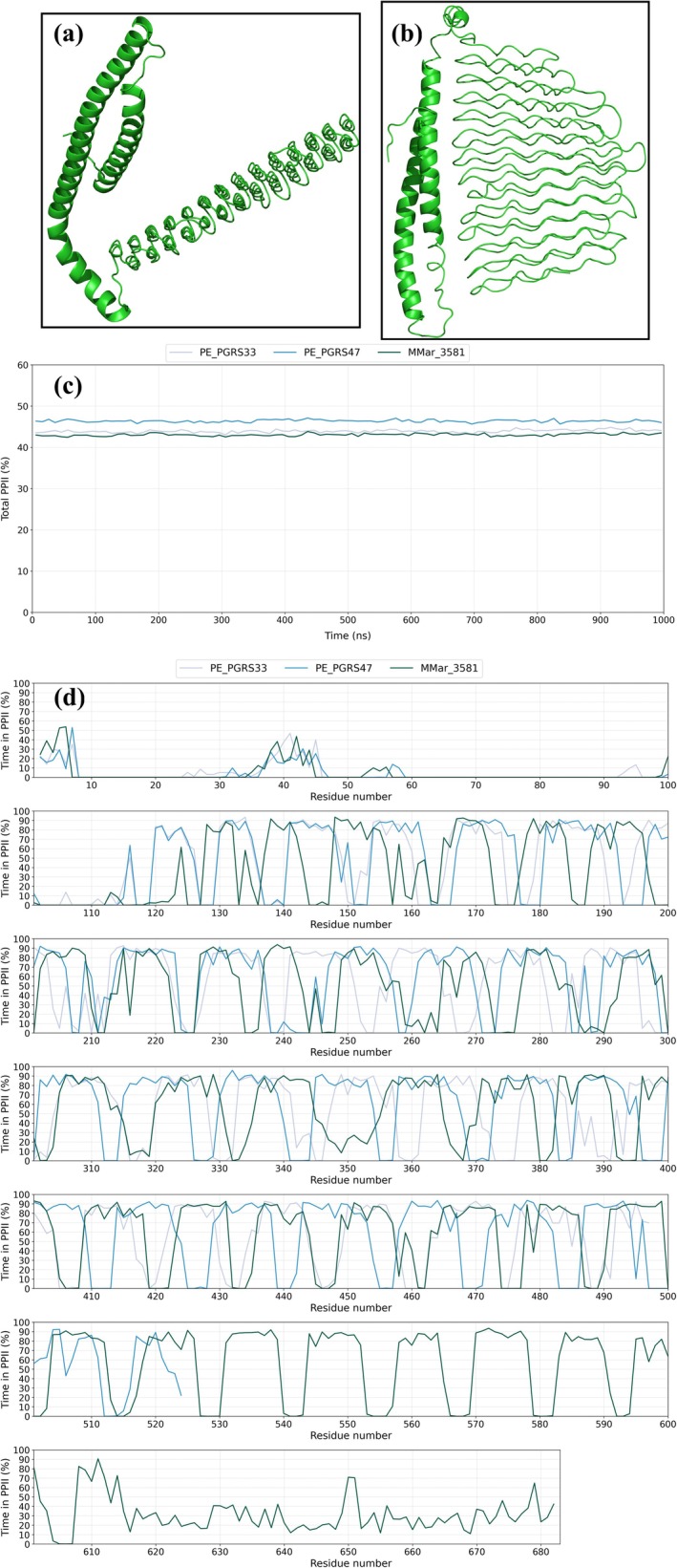
(a) AlphaFold Server structural model for *M. tuberculosis* PE_PGRS33 following equilibration at the beginning of the simulation (*t* = 0 ns) showing the α‐helical PE domain (*left*) and the PGRS domain (*right*) consisting of a bundle of 27 PPII helices arranged in a bilayer. By the end of the simulation (b) (*t* = 1000 ns), these structures remain folded and the PGRS domain has swung around to allow its hydrophobic loops, which have been proposed to surf the outer bacterial membrane as proposed by Berisio and Delogu (2022), to rest against the PE domain. (c) Percent of PPII conformation for *M. tuberculosis* PE_PGRS33 (gray), PE_PGRS47 (blue) and *M. marinum* MMar3581 (teal). (d) Per‐residue average PPII populations over the course of the simulations for *M. tuberculosis* PE_PGRS33 PE_PGRS33 (gray), PE_PGRS47 (blue) and *M. marinum* MMar3581 (teal).

The residue‐resolved PPII population profiles reveal clear differences among the proteins analyzed and provide a useful framework for assessing their conformational behavior during MD simulations (Figures 4 and 5). Residues displaying high PPII occupancy over the simulation time can be interpreted as belonging to persistent PPII‐rich segments, and when these high‐occupancy regions extend over long glycine‐rich stretches, they are consistent with the formation of stable PPII bundles. By contrast, residues with low or fluctuating PPII percentages are more indicative of transient, weakly populated PPII helices. Importantly, the PPII populations are not randomly distributed along the sequences; rather, each protein displays a characteristic and reproducible pattern of high‐ and low‐PPII regions. This interpretation is reinforced by two additional independent simulations for each protein, which yielded highly similar residue‐resolved PPII profiles and reproduced the same overall sequence‐dependent trends (Figures S2–S15). Importantly, these simulations were not selected arbitrarily, but were designed to test PPP‐derived hypotheses in proteins representing different predicted PPII regimes, from strongly glycine‐rich bundle‐like candidates to more weakly populated or cryptic PPII‐prone segments. In this sense, AF PSD provided the initial structural models, whereas PPP supplied the sequence‐based rationale for selecting and comparing the systems. Together, these analyses show that the starting models are informative for probing PPII‐rich conformational behavior, while also allowing the simulations to discriminate between stable, persistent PPII‐rich states and more heterogeneous or weakly populated conformational ensembles.

A marked contrast is observed between the plant glycine‐rich protein (Figure 4 and Figures S2–S4, S8–S10) and the mycobacterial PE_PGRS proteins (Figure 5 and Figures S14–S22). The kidney bean cell wall protein, wild carrot embryos, and RIPK3 display rather heterogeneous PPII populations (Figure 4 and Figures S2–S4, S8–S13). The kidney bean cell wall protein exhibits an overall fragmented profile dominated by low and moderate PPII populations (Figure 4 and Figures S2–S4), with only scattered local maxima across its glycine‐rich regions. Notably, the AF PSD model predicts, albeit with low confidence, a double β‐sheet arrangement for these glycine‐rich stretches. However, the MD results do not support the persistence of a stable β‐rich architecture and instead point to a conformationally heterogeneous ensemble in which weakly populated PPII‐like segments are transiently sampled. Wild carrot embryo protein also shows predominantly low‐to‐moderate PPII helical content (Figure 4 and Figures S8–S10), with most residues remaining below ~50% and only isolated positions reaching higher occupancies. Even in the glycine‐rich C‐terminal half, the PPII conformation is discontinuous and heterogeneous, with no consolidated PPII helical bundle. RIPK3 displays an even more fragmented behavior (Figure 4 and Figures S11–S13), with generally low PPII occupancies interrupted by sparse peaks at individual residues or short sequence patches. This pattern is more consistent with transient or partly populated PPII‐like conformations than with an extended, stable bundle. Thus, for these systems, the simulations support the presence of local PPII propensity but not a robust, consistently maintained PPII‐bundle architecture.

By contrast, the mycobacterial proteins PE_PGRS33, PE_PGRS47, and MMar_3581 display extended regions with very high PPII populations, frequently in the ~80%–90% range, especially throughout their glycine‐rich PGRS domains (Figure 5 and Figures S14–S22). These profiles are strongly suggestive of stable and persistent PPII‐rich assemblies, fully consistent with the presence of glycine‐rich PPII bundles. In PE_PGRS33 (Figure 5 and Figures S14–S16), after the relatively low‐PPII N‐terminal PE domain, which is known to adopt long α‐helices, the central and C‐terminal portions show broad plateaux of high PPII occupancy separated by short interruptions corresponding to loops, indicating a largely stable bundled state. PE_PGRS47 behaves similarly (Figure 5 and Figures S17–S19), with its N‐terminal PE domain remaining weakly PPII‐populated but the long glycine‐rich region from approximately residue 100 onward showing repeatedly high and sustained PPII values, again consistent with a robust bundled architecture. MMar_3581 exhibits the strongest overall tendency toward persistent PPII (Figure 5 and Figures S20–S22), with most of its long central glycine‐rich domain remaining at high PPII occupancy over extended sequence intervals, interrupted only by local decreases related to turns. Taken together, these three proteins provide the clearest computational signature of stable glycine‐rich PPII bundles among the systems studied.

As a negative control for sequence specificity, and using PE_PGRS33 as a representative example of a PE_PGRS protein displaying strong PPII‐rich bundle‐like behavior, we generated a scrambled version of its TRL2‐interacting domain (residues 140–260), predicted its structure with the AlphaFold Server, and subjected the resulting model to MD simulation. Unlike the native sequence, the scrambled construct failed to sustain extended regions of high PPII occupancy (Figure 4 and Figures S5–S7). Rather, it displayed a discontinuous residue‐resolved profile dominated by low‐to‐moderate PPII populations, with only isolated peaks and no long plateaux consistent with stable PPII‐rich segments or bundle‐like organization. Because the scrambled sequence preserves overall composition while disrupting the native residue order, these findings indicate that the persistent PPII propensity of the native PE_PGRS33 segment is sequence‐dependent and cannot be explained as a nonspecific consequence of glycine enrichment alone.

These MD results identified segments in the PGRS domains that adopt stable PPII helices and others forming non‐PPII helical turn or loop segments. To more finely test PPP, we measured the number of matches found by PPP for segments in the *M. tuberculosis* PGRS33 domain that adopt stable PPII helices versus loop or turn segments. Many more are obtained for the former, and this suggests that PPP distinguishes between these two classes (Table S1).

### 2.4 Experimental validation of PPII helix conformation in human RIPK3

Among the systems identified by the PPP server, human RIPK3 was selected for experimental characterization due to its high biological relevance and the extensive functional knowledge available for this protein (Pham et al., 2023; Wu et al., 2021). The identification by PPP of proline‐rich and glycine‐rich segments with strong PPII propensity in the vicinity of the RHIM region, otherwise regarded as disordered, raises the possibility that PPII conformations may influence the structural organization or regulatory dynamics of RIPK3 signaling complexes. In addition, RIPK3 has been extensively studied structurally and biochemically, facilitating the design and interpretation of experimental assays aimed at validating the structural predictions generated by the PPP server. Using a minimum subset length of nine residues and a similarity threshold of 66%, two segments in the “pre‐RHIM” motif exceeded the web server's empirical PPII propensity cutoff, as previously mentioned.

**FIGURE 6:**
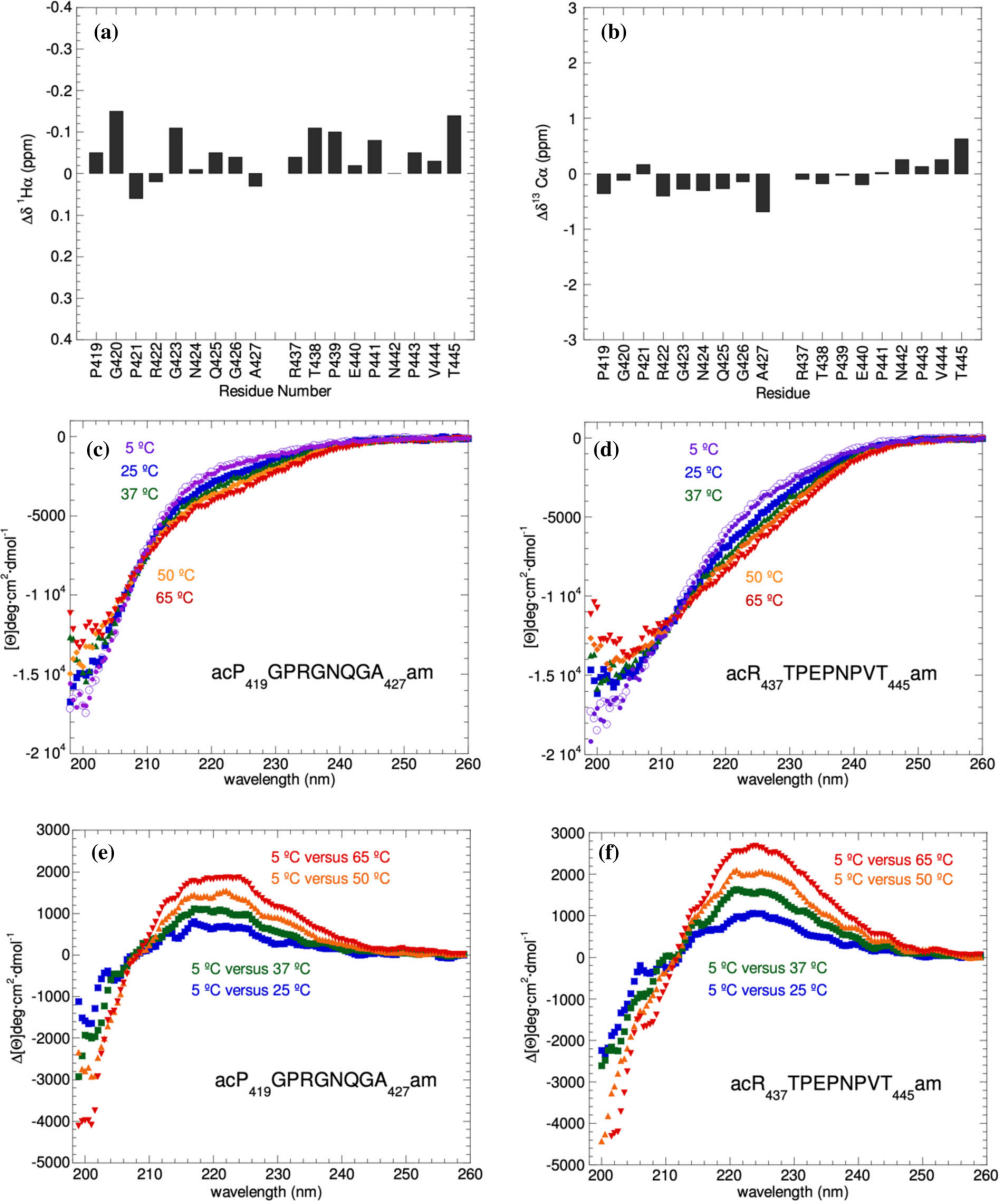
NMR and CD data confirm partial PPII helices in RIPK3 RHIM flanking segments. (a) Small ^1^H_α_ and (b) ^13^C_α_ conformational chemical shifts rule out the presence of significant populations of α‐helical and β‐strand conformations. (c,d) Far‐UV CD spectra of RIPK residues 419–427 (c) and residues 437–445 (d) recorded at 5 (purple), 25 (blue), 37 (green), 50 (orange) and 65°C reveal heat‐induced spectral changes. (e,f) Far‐UV CD temperature difference spectra of RIPK residues 419–427 (e) and 437–445 (f); the maximum near 223 nm is a hallmark of PPII helices.

To validate these specific PPP‐based predictions experimentally, we characterized the corresponding peptides, acP_419_GPRGNQGA_427_am and acR_437_TPEPNPVT_445_am. Conformational chemical shifts (Δδ) of ^1^Hα and ^13^Cα for both peptides are small (Figure 6a,b), indicating an absence of stable α‐helical or β‐strand conformations and an ensemble consistent with PPII helix or statistical coil. Far‐UV CD spectra showed no characteristic negative bands for α‐helix (208, 222 nm) or β‐strand (217 nm) (Figure 6c,d). Difference spectra obtained by subtracting higher temperature spectra from those at 5°C revealed a maximum between 220 and 230 nm, characteristic of PPII helical structure that converts to statistical coil upon heating (Figure 6e,f) (Kjaergaard et al., 2010). Taken together, the NMR and CD data support the presence of non‐α, non‐β conformational ensembles with measurable PPII character in both peptides. Additional CD experiments, performed to assess reproducibility and uncertainties, are shown in Figure S23 and Table S2. Quantitative analysis of all the CD data estimated significant PPII helix populations at 5°C (~30% for acPGPRGNQGAam and ~15% for acRTPEPNPVTam) (Park et al., 1997). These values are qualitatively consistent with the MD simulations of the corresponding RIPK3 C‐terminal region, which likewise support appreciable PPII populations in both segments. In the first MD simulation, the mean PPII populations were ~32% for residues 419–427 (PGPRGNQGA) and ~22% for residues 437–445 (RTPEPNPVT) (Figure 4), and comparable overall values were obtained in the two additional independent simulations (Figures S11–S13). Although the simulated populations are slightly higher overall, this difference is not unexpected given that the MD results were obtained for the larger protein fragment, whereas the CD measurements were performed on isolated capped peptides under different experimental conditions. The convergence of CD, NMR, and MD data therefore provides strong support for the PPP prediction and demonstrates that RIPK3's intrinsically disordered C‐terminus harbors discrete, partially populated PPII conformations flanking its RHIM core.

## 3 DISCUSSION

The PPP webserver provides a fast and sensitive way to identify sequences that may potentially adopt polyproline II helices. Compared to previously published methods, PPP is particularly effective at detecting glycine‐rich segments that other tools overlook. Some algorithms capable of predicting PPII helices are not available via web servers and require code installation and computational skills, so we do not include them in our comparative analysis. Instead, we focused on the four web‐based tools currently operational: PolyprOnline, DSSP 4, PPIIPRED and DynaMine, available through the Bio2Byte tools platform. PolyprOnline and DSSP 4 require a PDB file from the RCSB PDB or a user‐provided structure; thus, they cannot predict them based on a query sequence. In contrast, PPIIPRED predicts PPII secondary structure from the amino acid sequence using a bidirectional recurrent neural network, providing residue‐level scores from 0.00 to 1.00. DynaMine rapidly predicts α‐helix, β‐sheet, coil and PPII conformational propensities, also returning residue‐level scores from 0.00 to 1.00. For the two RIPK3 pre‐RHIM segments identified by our PPP server as likely PPII helices (PGPRGNQGA, residues 419–427; and RTPEPNPVT, residues 437–445), PPIIPRED and DynaMine yielded low average scores of 0.142 and 0.208, respectively, for these same sequences.

A broader benchmarking across all positive examples considered here further highlights the compositional versatility of PPP. This set included the experimentally validated RIPK3 pre‐RHIM motifs, the glycine‐rich PPII‐bundle proteins sfAFP, S16 gp38, and ALK, and two structurally established proline‐rich reference systems, (PPG)_10_ (RCSB PDB ID 1K6F) (Berisio et al., 2002) and PRAD (RCSB PDB ID 1VZJ) (Dvir et al., 2004). The latter are especially informative because they represent classical PPII‐rich architectures: a collagen model with three PPII helices wrapped around a common axis forming its well‐established triple helix (Berisio et al., 2002), and the proline‐rich attachment domain that forms a PPII helix around which four WAT chains assemble in the acetylcholinesterase tetramerization complex (Dvir et al., 2004). PPIIPRED performed best in these canonical proline‐rich contexts, most notably the collagen model (0.294) and PRAD (0.419), and it also showed stronger mean signal in RIPK3 pre‐RHIM segments (0.142) than in most glycine‐rich bundle‐forming proteins, particularly in S16 gp38 (0.018), sfAFP (0.085), and ALK (0.023) (Table 1). By contrast, DynaMine returned low mean PPII scores across all systems examined, including sfAFP (0.226), S16 gp38 (0.193), and ALK (0.176) (Table 1). Even in the proline‐rich reference systems and in RIPK3, the corresponding mean values remained relatively low, namely collagen (0.357), PRAD (0.185), and RIPK3 pre‐RHIM segments (0.208), indicating that DynaMine captures only modest PPII tendencies even in validated PPII‐positive regions. Nevertheless, in all six systems the dominant DynaMine assignment remained coil rather than PPII, indicating that these PPII tendencies are not cleanly separated from a broader coil‐like interpretation. PPP, in contrast, consistently recovered proline‐rich, glycine‐rich, and compositionally mixed PPII‐positive systems, including canonical proline‐rich motifs, experimentally established glycine‐rich bundles, and the two RIPK3 segments validated here (Table 1). The small fraction of experimentally validated residues not recovered by PPP most likely reflects the fact that some of the corresponding PPII helices do not fall within the canonical dihedral‐angle ranges used by the program for the analysis of RCSB PDB structures (see Materials and Methods).

**TABLE 1:** Benchmarking of PPP, PPIIPRED, and DynaMine across experimentally supported PPII‐positive systems.

Protein	Context	Validated PPII segments	PPP coverage (%)	PPIIPRED mean score	DynaMine PPII mean score	DynaMine dominant class
RIPK3	Pro/Gly motifs in IDR	419–427; 437–445	100.0	0.142	0.208	Coil
sfAFP	Gly‐rich bundle	2–11; 15–24; 29–39; 44–54; 59–68; 72–81	100.0	0.085	0.226	Coil
S16 gp38	Gly‐rich bundle	115–121; 124–131; 151–158; 166–172; 174–180; 185–190; 193–198; 205–210; 213–218; 222–230	94.3	0.018	0.193	Coil
ALK	Gly‐rich bundle	740–747; 751–757; 776–782; 816–824; 841–848; 869–875; 878–882; 891–894; 899–906; 914–920; 922–927; 931–935; 938–943; 950–956	90.4	0.023	0.176	Coil
(PPG)_10_	Pro‐rich collagen‐like	Whole sequence	100.0	0.294	0.357	Coil
PRAD	Pro‐rich isolated	Whole sequence	100.0	0.419	0.185	Coil

*Note*: The set includes the two RIPK3 pre‐RHIM segments validated in this work, the glycine‐rich PPII‐bundle proteins sfAFP, S16 gp38, and ALK, and the proline‐rich reference systems PRAD and collagen (PPG)_10_. For RIPK3, sfAFP, S16 gp38, and ALK, mean values were calculated over the experimentally validated sequence segments known to adopt PPII helices (Rodríguez & Laurents, 2024). For collagen (PPG)_10_ and PRAD, the whole sequence was used as the validated PPII‐positive fragment (Berisio et al., 2002; Dvir et al., 2004). PPP coverage indicates the percentage of validated residues recovered by PPP. PPIIPRED and DynaMine values correspond to the mean score over the validated segments(s), and the dominant DynaMine class indicates the secondary‐structure state most strongly assigned over those same regions.

Extending this analysis, a set of homopeptides and protein segments reported by experimental approaches to adopt, at least partially, PPII helical conformations were used to test the performance of PPP alongside Dynamine and PPIIPred as well as AlphaFold PSD and Server (Table S3). In all cases, PPP found similar sequences adopting bona fide PPII helices in protein structures, while in many cases AlphaFold did not predict PPII conformations or predicted them with low confidence. Dynamine and PPIIPred generally predicted that these segments adopt moderate populations of PPII conformations, with Dynamine scoring glycine‐rich segments higher than PPIIPred and PPIIPred scoring proline‐rich segments higher. Interestingly, the number of RCSB PDB matching segments found by PPP correlates approximately with the percent PPII helical population predicted by Dynamine and PPIIPred.

Taken together, these comparisons suggest that PPIIPRED is most sensitive to localized proline‐rich PPII‐like patterns, DynaMine captures broader PPII tendencies but does not clearly resolve them from coil, and PPP is the most compositionally versatile and structurally informative predictor among the methods examined. Although PolyprOnline and DSSP 4 provide a valuable structure‐based reference for curated PPII assignments, it was not included in the main benchmarking because, unlike PPP, it requires an existing 3D structure and therefore does not address de novo sequence‐based prediction. Nevertheless, our benchmark includes several proteins with experimentally determined structures and established PPII‐rich regions, thereby incorporating structurally validated positive cases into the comparison.

Other online tools can provide indirect support for segments with possible PPII character by identifying regions predicted to interact with PPII‐recognizing domains. For instance, MoDPepInt (Kundu et al., 2014) predicts binding partners for modular protein domains, including SH2, SH3, and PDZ, while LMDIPred (Sarkar et al., 2018) identifies short linear motifs predicted to interact with SH3, WW, and PDZ domains. For the RIPK3 sequence, MoDPepInt highlights a potential SH3‐binding stretch (PVLLHRDLKPSNVLL, residues 136–150). LMDIPred identifies up to 30 possible SH3‐binding motifs and 123 WW‐binding motifs, many of which overlap. Among these, residues 417–422 and 438–443 (SH3 option) and 428–433 and 438–443 (WW option) overlap with segments predicted by PPP. Although these tools are restricted to six‐residue motifs, reflecting the typical size of peptides binding to SH3 and WW domains, their partial agreement with the PPP‐predicted regions provides additional support for the functional plausibility of these segments.

Compared to existing secondary‐structure predictors, which may overlook or misclassify glycine‐rich motifs as random coil, our server incorporates specialized PPII propensity scales and context‐dependent scoring derived from experimental datasets. The correspondence between in silico scores and biophysical observables in the RIPK3 case underscores the utility of explicitly modeling PPII helices when analyzing low‐complexity or glycine/proline‐rich regions. This conclusion is further strengthened by the experimental and computational validation of RIPK3. The two pre‐RHIM peptides identified by PPP showed small conformational chemical shifts and CD spectra lacking signatures of stable α‐helical or β‐strand structure, whereas temperature‐dependent difference spectra supported the presence of partially populated PPII conformations. Quantitative analysis of the CD signal estimated PPII populations of approximately 30% for acPGPRGNQGAam and 15% for acRTPEPNPVTam at 5°C. These values are qualitatively consistent with the MD simulations of the corresponding RIPK3 C‐terminal region, which likewise support appreciable PPII populations in both segments. Thus, PPP does not merely recover generic low‐complexity motifs, but rather identifies sequence elements with genuine PPII‐forming potential in a region otherwise regarded as disordered. More importantly, the RIPK3 case shows that local PPII‐prone elements can flank an amyloidogenic core and may therefore contribute to the conformational organization or regulatory dynamics of a functional amyloid assembly pathway.

The broader set of MD simulations extends this conclusion beyond RIPK3 and shows that, although PPP identified all of these proteins as containing PPII‐prone segments, their subsequent conformational behavior is not uniform. Glycine‐rich PE_PGRS‐like sequences such as PE_PGRS33, PE_PGRS47, and MMar_3581 displayed long‐lived, highly populated PPII‐rich regions consistent with persistent bundle‐like architectures. By contrast, the plant glycine‐rich proteins and RIPK3 retained lower and more heterogeneous PPII populations, compatible with transient or only partially formed PPII helices rather than fully developed assemblies. The negative‐control simulation on the scrambled PE_PGRS33 segment further showed that glycine richness alone is insufficient to reproduce the sustained high‐PPII signature of the native sequence, indicating that persistent PPII‐rich conformational behavior depends on the specific native residue order. Importantly, these results show that PPP is useful not only for detecting proteins that sustain persistent PPII‐rich bundle‐like architectures, but also for identifying systems that contain more local or only partially populated PPII helices. In this context, AF PSD models were useful as structural starting points, whereas MD provided the additional discriminatory power needed to distinguish between these different conformational outcomes of PPP‐predicted PPII propensity.

PPP provides an important advantage complementary to global AF PSD structural models by specifically highlighting PPII‐prone sequence segments in regions that may otherwise appear merely disordered or receive only low‐confidence alternative assignments, such as β‐sheet‐like conformations. However, because PPP relies on a reference database derived from the RCSB PDB, it may underrepresent highly dynamic or weakly populated PPII conformations in IDRs, which constitutes an inherent limitation of the method. Even considering this limitation, the RIPK3 results show that PPP can still identify biologically relevant PPII‐prone segments within a region that is globally regarded as disordered. Furthermore, PPP does not by itself establish the precise population, supramolecular organization, or functional role of those elements in their native protein context. Residue‐resolved PPII populations obtained from MD simulations report local backbone geometry and persistence, but do not by themselves establish the full supramolecular organization or functional relevance of a given segment. At the same time, the isolated peptides of the NMR and CD validation may not fully recapitulate the conformational ensembles present in the full‐length protein or within multicomponent necrosome assemblies. Future experimental corroboration is needed to confirm these PPII helices' formation in the context of the intact protein, assess their role in homo‐ and heteromeric amyloid formation, and explore how post‐translational modifications modulate PPII stability.

In conclusion, the PPP server presented in this work reliably predicts PPII helical segments in supposedly disordered protein regions, validated here by CD and NMR for RIPK3 residues 419–427 and 437–445. These newly identified PPII helices flank the RHIM amyloid core. Considering that segments with a high propensity to adopt PPII helix flank the amyloid forming regions of huntingtin (Bhattacharyya et al., 2006) and the prion protein (Kraus et al., 2015) and hinder their aggregation, it is plausible that these partly populated PPII helices of RIPK3 modulate RHIM‐mediated assembly, influencing nucleation and monomer recruitment. This approach can be extended proteome‐wide to uncover PPII elements in various. More generally, PPP provides an interpretable framework for identifying PPII‐prone segments across proteomes and for prioritizing targets for downstream structural and biophysical validation. This approach can be extended proteome‐wide to uncover PPII elements in various low‐complexity domains, offering insights into mechanisms of protein–protein interaction, assembly regulation, and potential therapeutic targeting of amyloid‐driven signaling pathways.

## 4 MATERIALS AND METHODS

### 4.1 Computational overview

The PPP web server functions by prompting the user for an email‐supplied code, followed by query sequence entry (standard one‐letter amino acid codes). The first residue number can be specified, defaulting to one. Users select a minimum segment length (3, 6, 9, or 12 residues), corresponding to one to four turns of a PPII helix. This choice controls the sliding‐window search, with longer lengths favoring detection of more extended, stable PPII helices, and shorter lengths increasing sensitivity. The default is six residues. A similarity threshold (0–100%) is also selected; higher settings yield fewer high‐confidence matches. The default value is 66%.

The server's Python 3 backend, integrated with an HTML 5 and PHP 8 web interface, first validates the query and applies user‐chosen parameters. It compares every qualifying subsequence against a curated database of PPII helix fragments from the RCSB PDB using ungapped alignments between segments of equal length. Similarity is quantified as percent sequence identity across the full segment length, and only matches meeting or exceeding the user‐defined threshold are retained. The database was constructed by parsing over 230,000 entries (up to December 2024), identifying residues with dihedral angles Φ ∈ [−95°, −55°] and Ψ ∈ [125°, 175°]. To maintain currency as the RCSB PDB expands, the database is updated every 18 months. There is some debate over the boundaries of the PPII region of the Ramachandran diagram (Hollingsworth & Karplus, 2010). The cutoffs chosen here define a deliberately narrower and thus more conservative PPII region than the broader windows used by established tools such as PolyprOnline (Chebrek et al., 2014), favoring specificity in the identification of PPII‐like segments. For glycine residues, the Ψ angle range was extended by ±10° to account for their greater conformational flexibility (Hollingsworth & Karplus, 2010), which manifests itself in canonical structures composed of PPII helices such as the collagen triple helix. Contiguous stretches of at least three such residues were retained. No additional filtering based on global structure‐quality metrics, such as resolution, R‐factors, or confidence scores, was applied to the RCSB PDB‐derived database. This was a deliberate choice, because PPII helices are often transient and associated with flexible or poorly resolved regions, and stringent global quality filters could therefore bias the database against biologically relevant PPII‐containing segments.

PPP then scans the sequence for recurring sequence motifs associated with PPII‐related conformations, including PPX, PXX, GGX, and GXX, which must appear at least twice consecutively, and PX, that must appear at least three times consecutively for reporting (Table 2). X denotes any amino acid residue. To avoid overinterpreting very short motifs, the recurrence criteria were selected so that the reported patterns span at least six residues, that is, two turns of a PPII helix, which is a more plausible minimum length for a structurally meaningful PPII‐prone segment. The server outputs a report listing those motifs associated with PPII propensity, together with query matches to structurally identified PPII‐like segments in the RCSB PDB. For each match, the output includes the RCSB PDB ID, its reference PPII fragment, and the matching segment in the query sequence (start‐end positions, length, and identity percentage). This provides complementary views of internal sequence patterns and external alignment with experimentally validated PPII segments.

**TABLE 2:** Sequence motifs associated with PPII propensity considered by PPP.

Motif	PPII helical structure
PPX	Uninterrupted PPII rods rich in proline residues that function as rigid spacers separating domains (Morgan & Rubenstein, 2013; Williamson, 1994)
PXX	The PXXP‐like recognition helix that fits into SH3, WW, and EVH1 domains (Kay et al., 2000; Macias et al., 2002; Meirson et al., 2020)
PX	Extended PPII helices that serve as structural stalks in some bacterial surface adhesins (Larson et al., 2010; Tamborrini et al., 2015)
GXX	Each chain is a PPII helix and three chains wind together into the well‐known collagen triple helix (Bella & Berman, 1996)
GGX	Glycine‐rich PPII bundles found in antifreeze‐type proteins or the hexagonal packing of polyglycine II (Rich & Crick, 1955; Treviño et al., 2018)

*Note*: X denotes any amino acid residue. Recurrence thresholds were chosen so that reported motifs cover at least six residues, equivalent to about two turns of a PPII helix. Accordingly, PPX, PXX, GGX, and GXX motifs must appear at least twice consecutively, whereas PX motifs must appear at least three times consecutively. This criterion was introduced to reduce false positives and to prioritize sequence patterns more likely to correspond to structurally meaningful PPII‐prone segments.

### 4.2 Molecular dynamics simulations

The protein force field CHARMM36m (Huang et al., 2017), within the CHARMM36 2022 release, was used for its ability to accurately simulate ordered and disordered protein conformations and their interconversions, as well as its proven performance in previous studies addressing the modeling of proline‐rich PPII helices and glycine‐rich PPII bundles (Hill & Welch, 2022; Olson, 2018). A total of twenty‐one 1‐μs different runs, three per each protein under study, were run with different initial velocities using the CHARMM‐modified TIP3P water model at 300 K and 1 atm. We embedded the systems in the most volume‐efficient (optimal) dodecahedral periodic simulation box, leaving a minimum solute–box distance of 1.5 nm in all directions, and added KCl to neutralize the net charge and reach a final salt concentration of 150 mM. After steepest descent energy minimization, each system was pre‐equilibrated for 1 ns under NVT and then 1 ns under the NpT ensemble, using a modified Berendsen thermostat (Berendsen et al., 1984) and a Parrinello‐Rahman barostat (Parrinello & Rahman, 1981). The productions were run in the NpT ensemble, controlling the temperature and pressure using the same algorithms as during the equilibrium phase. The electrostatic interactions were calculated using the Particle Mesh Ewald algorithm, with a 1.2 nm cut‐off radius (Darden et al., 1993). The cut‐off for van der Waals forces was also 1.2 nm. The integration time step was 2 fs as enabled by the LINCS algorithm (Hess et al., 1997). All MD simulations were performed using GROMACS version 2025.2 (Abraham et al., 2015). The trajectories were visualized using PyMOL 2.3.0 (DeLano, 2002). The dihedral angles considered to classify residues within the PPII conformation were the same as when analyzing the RCSB PDB entries: Φ ∈ [−95°, −55°] and Ψ ∈ [125°, 175°], with an extended Ψ range of 10° for glycine.

### 4.3 Peptide synthesis and purification

Peptides acPGPRGNQGAam and acRTPEPNPVTam were synthesized and purified by Caslo (Denmark). The letters “ac” and “am” correspond to N‐terminal acetyl and C‐terminal amide groups, respectively, which were added to maintain the termini uncharged, as they would be in the context of the full length protein. The final purity was over 99%. Peptide identity was confirmed by mass spectrometry and NMR spectroscopy.

### 4.4 Circular dichroism

Far‐UV CD spectra were recorded on a Jasco 810 spectropolarimeter with a Peltier temperature control unit. Spectra were collected from 190 to 260 nm (or 198–260 nm) at a scan speed of 50 nm/min, 1.2–1.67 nm bandwidth, using 0.275 mM peptide in 10 mM K_2_HPO_4_ buffer (pH 6) in a 1 mm pathlength cuvette. The concentration of the peptides was estimated by weight and measured more precisely by comparing peptide 1D ^1^H NMR peak integrals to 1D ^1^H NMR peak integrals of a reference of known concentration. Eight to 10 scans were averaged per spectrum, and a buffer spectrum was subtracted. Thermal unfolding experiments were monitored by CD at 217 nm from 0°C to 65°C at a 1°C/min heating rate and 1.0 nm bandwidth. Reversibility was assessed by recording spectra at 0°C before and after denaturation. The percentage of PPII helix was estimated using the empirical equation of Stellwagen and coworkers (Park et al., 1997).

$$\%\text{PPII helix}=100\cdot \frac{\;{\left[\Theta \right]}_{\max}+5560\;\deg\cdot {\mathrm{cm}}^{2}\cdot {\text{dmol}}^{-1}}{15140\;\deg\cdot {\mathrm{cm}}^{2}\cdot {\text{dmol}}^{-1}}$$

where [Θ]_max_ is the maximum molar ellipticity between 210 and 230 nm in deg·cm^2^·dmol^−1^. Based on an uncertainty of about 500 deg·cm^2^·dmol^−1^ in the CD ellipticity values (Park et al., 1997) and differences in the % PPII helix estimated by CD spectroscopy (Gates et al., 2017) as compared to x‐ray crystallography (Pentelute et al., 2008) and NMR methods (Treviño et al., 2018), the uncertainty in the % PPII helix calculated from CD is about 6%.

### 4.5 Nuclear magnetic resonance

NMR spectra, including 2D ^1^H—^1^H COSY, TOCSY, NOESY, and 2D ^1^H—^13^C HSQC and ^1^H—^15^N HSQC, were recorded on 1–3 mM peptide samples in 10 mM K_2_HPO_4_ buffer (pH 6) at 5°C on a 600 MHz (^1^H) Bruker AvanceNeo spectrometer. The pH in situ was confirmed by measurement of the ^31^P chemical shift of the phosphate buffer (Pantoja‐Uceda & Laurents, 2025) using the on‐line webserver: https://rmni.iqf.csic.es/software/31phnmr/. Spectral parameters are summarized in Table S4. Spectra were assigned with the aid of NMRFAM‐SPARKY (Lee et al., 2015) and analyzed to obtain ^1^H_α_ and ^13^C_α_ chemical shifts. These chemical shifts were compared to reference values for a structureless peptide for the same temperature and pH value (Kjaergaard et al., 2011). The differences, also known as the conformational chemical shifts (δΔ), were used to discriminate between α‐helical, β‐strand, and statistical coil/PPII conformations (Figure 4a,b).

#### AI TOOLS

ChatGTP was used to help write a first draft of the Computational Overview section, which was later revised by the authors.

## AUTHOR CONTRIBUTIONS

**Douglas V. Laurents:** Project administration; writing – review and editing; validation; conceptualization; investigation; funding acquisition; formal analysis; visualization; supervision; resources; writing – original draft. **Rubén López‐Sánchez:** Conceptualization; investigation; methodology; validation; writing – original draft; writing – review and editing; software; data curation; formal analysis; visualization. **Miguel Mompeán:** Conceptualization; investigation; methodology; validation; writing – review and editing; software; formal analysis; supervision; resources; funding acquisition; visualization; writing – original draft; data curation. **David Pantoja‐Uceda:** Methodology; software; data curation; resources; supervision; conceptualization; investigation; validation; writing – review and editing; formal analysis; visualization.

## Supporting information


**Figure S1.** AlphaFold Server structural model of a bacteriophage glycine‐rich protein linked to a polyglycine peptide. (A) Designed variant of a PPII helical bundle domain from the Salmonella phage tail spike protein bound noncovalently to a polyglycine peptide on the left. The high and moderately high confidence of these predictions are colored blue and cyan, respectively, according to the AlphaFold pIDDT score. (B) Alternative perspective of A. In the sequence shown below the structural models, glycine residues are highlighted in yellow.
**Figure S2.** Residue‐resolved PPII populations in the 1‐μs MD simulation of the kidney bean cell wall glycine‐rich protein. Values represent the fraction of simulation time during which each residue adopted PPII backbone dihedral angles, starting from the corresponding AlphaFold Protein Structure Database model (AF‐P10496‐F1‐v6). Glycine residues are highlighted in yellow. Results from two additional 1 μs MD simulations are shown in Figures S3 and S4.
**Figure S3.** Residue‐resolved PPII populations in the second 1‐μs MD simulation of the kidney bean cell wall glycine‐rich protein. Values represent the fraction of simulation time during which each residue adopted PPII backbone dihedral angles, starting from the corresponding AlphaFold Protein Structure Database model (AF‐P10496‐F1‐v6). Glycine residues are highlighted in yellow. Results from two additional 1 μs MD simulations are shown in Figures S2 and S4.
**Figure S4.** Residue‐resolved PPII populations in the third 1‐μs MD simulation of the kidney bean cell wall glycine‐rich protein. Values represent the fraction of simulation time during which each residue adopted PPII backbone dihedral angles, starting from the corresponding AlphaFold Protein Structure Database model (AF‐P10496‐F1‐v6). Glycine residues are highlighted in yellow. Results from two additional 1 μs MD simulations are shown in Figures S2 and S3.
**Figure S5.** Residue‐resolved PPII populations in the 1‐μs MD simulation of the scrambled TRL2 interacting domain of PE_PGRS33 from *Mycobacterium tuberculosis*. Values represent the fraction of simulation time during which each residue adopted PPII backbone dihedral angles, starting from the generated AlphaFold Server model. Glycine residues are highlighted in yellow. Results from two additional 1 μs MD simulations are shown in Figures S6 and S7.
**Figure S6.** Residue‐resolved PPII populations in the second 1‐μs MD simulation of the scrambled TRL2 interacting domain of PE_PGRS33 from *Mycobacterium tuberculosis*. Values represent the fraction of simulation time during which each residue adopted PPII backbone dihedral angles, starting from the generated AlphaFold Server model. Glycine residues are highlighted in yellow. Results from two additional 1 μs MD simulations are shown in Figures S5 and S7.
**Figure S7.** Residue‐resolved PPII populations in the third 1‐μs MD simulation of the scrambled TRL2 interacting domain of PE_PGRS33 from *Mycobacterium tuberculosis*. Values represent the fraction of simulation time during which each residue adopted PPII backbone dihedral angles, starting from the generated AlphaFold Server model. Glycine residues are highlighted in yellow. Results from two additional 1 μs MD simulations are shown in Figures S5 and S6.
**Figure S8.** Residue‐resolved PPII populations in the 1‐μs MD simulation of the wild carrot embryo glycine‐rich protein. Values represent the fraction of simulation time during which each residue adopted PPII backbone dihedral angles, starting from the corresponding AlphaFold Protein Structure Database model (AF‐Q39691‐F1‐v6). Glycine residues are highlighted in yellow. Results from two additional 1 μs MD simulations are shown in Figures S9 and S10.
**Figure S9.** Residue‐resolved PPII populations in the second 1‐μs MD simulation of the wild carrot embryo glycine‐rich protein. Values represent the fraction of simulation time during which each residue adopted PPII backbone dihedral angles, starting from the corresponding AlphaFold Protein Structure Database model (AF‐Q39691‐F1‐v6). Glycine residues are highlighted in yellow. Results from two additional 1 μs MD simulations are shown in Figures S8 and S10.
**Figure S10.** Residue‐resolved PPII populations in the third 1‐μs MD simulation of the wild carrot embryo glycine‐rich protein. Values represent the fraction of simulation time during which each residue adopted PPII backbone dihedral angles, starting from the corresponding AlphaFold Protein Structure Database model (AF‐Q39691‐F1‐v6). Glycine residues are highlighted in yellow. Results from two additional 1 μs MD simulations are shown in Figures S8 and S9.
**Figure S11.** Residue‐resolved PPII populations in the 1‐μs MD simulation of human RIPK3. Values represent the fraction of simulation time during which each residue adopted PPII backbone dihedral angles, starting from the corresponding AlphaFold Protein Structure Database model (AF‐Q9Y572‐F1‐v6). Glycine residues are highlighted in yellow. Results from two additional 1 μs MD simulations are shown in Figures S12 and S13.
**Figure S12.** Residue‐resolved PPII populations in the second 1‐μs MD simulation of human RIPK3. Values represent the fraction of simulation time during which each residue adopted PPII backbone dihedral angles, starting from the corresponding AlphaFold Protein Structure Database model (AF‐Q9Y572‐F1‐v6). Glycine residues are highlighted in yellow. Results from two additional 1 μs MD simulations are shown in Figures S11 and S13.
**Figure S13.** Residue‐resolved PPII populations in the third 1‐μs MD simulation of human RIPK3. Values represent the fraction of simulation time during which each residue adopted PPII backbone dihedral angles, starting from the corresponding AlphaFold Protein Structure Database model (AF‐Q9Y572‐F1‐v6). Glycine residues are highlighted in yellow. Results from two additional 1 μs MD simulations are shown in Figures S11 and S12.
**Figure S14.** Residue‐resolved PPII populations in the 1‐μs MD simulation of PE_PGRS33 from *Mycobacterium tuberculosis*. Values represent the fraction of simulation time during which each residue adopted PPII backbone dihedral angles, starting from the corresponding AlphaFold Protein Structure Database model (AF‐P9WIF5‐F1‐v6). Glycine residues are highlighted in yellow. Results from two additional 1 μs MD simulations are shown in Figures S15 and S16.
**Figure S15.** Residue‐resolved PPII populations in the second 1‐μs MD simulation of PE_PGRS33 from *Mycobacterium tuberculosis*. Values represent the fraction of simulation time during which each residue adopted PPII backbone dihedral angles, starting from the corresponding AlphaFold Protein Structure Database model (AF‐P9WIF5‐F1‐v6). Glycine residues are highlighted in yellow. Results from two additional 1 μs MD simulations are shown in Figures S14 and S16.
**Figure S16.** Residue‐resolved PPII populations in the third 1‐μs MD simulation of PE_PGRS33 from *Mycobacterium tuberculosis*. Values represent the fraction of simulation time during which each residue adopted PPII backbone dihedral angles, starting from the corresponding AlphaFold Protein Structure Database model (AF‐P9WIF5‐F1‐v6). Glycine residues are highlighted in yellow. Results from two additional 1 μs MD simulations are shown in Figures S14 and S15.
**Figure S17.** Residue‐resolved PPII populations in the 1‐μs MD simulation of PE_PGRS47 from *Mycobacterium tuberculosis*. Values represent the fraction of simulation time during which each residue adopted PPII backbone dihedral angles, starting from the corresponding AlphaFold Protein Structure Database model (AF‐Q79FB3‐F1‐v6). Glycine residues are highlighted in yellow. Results from two additional 1 μs MD simulations are shown in Figures S18 and S19.
**Figure S18.** Residue‐resolved PPII populations in the second 1‐μs MD simulation of PE_PGRS47 from *Mycobacterium tuberculosis*. Values represent the fraction of simulation time during which each residue adopted PPII backbone dihedral angles, starting from the corresponding AlphaFold Protein Structure Database model (AF‐Q79FB3‐F1‐v6). Glycine residues are highlighted in yellow. Results from two additional 1 μs MD simulations are shown in Figures S17 and S19.
**Figure S19.** Residue‐resolved PPII populations in the third 1‐μs MD simulation of PE_PGRS47 from *Mycobacterium tuberculosis*. Values represent the fraction of simulation time during which each residue adopted PPII backbone dihedral angles, starting from the corresponding AlphaFold Protein Structure Database model (AF‐Q79FB3‐F1‐v6). Glycine residues are highlighted in yellow. Results from two additional 1 μs MD simulations are shown in Figures S17 and S18.
**Figure S20.** Residue‐resolved PPII populations in the 1‐μs MD simulation of Mmar_3581 from *Mycobacterium marinum*. Values represent the fraction of simulation time during which each residue adopted PPII backbone dihedral angles, starting from the corresponding AlphaFold Protein Structure Database model (AF‐B2HL21‐F1‐v6). Glycine residues are highlighted in yellow. Results from two additional 1 μs MD simulations are shown in Figures S21 and S22.
**Figure S21.** Residue‐resolved PPII populations in the second 1‐μs MD simulation of Mmar_3581 from *Mycobacterium marinum*. Values represent the fraction of simulation time during which each residue adopted PPII backbone dihedral angles, starting from the corresponding AlphaFold Protein Structure Database model (AF‐B2HL21‐F1‐v6). Glycine residues are highlighted in yellow. Results from two additional 1 μs MD simulations are shown in Figures S20 and S22.
**Figure S22.** Residue‐resolved PPII populations in the third 1‐μs MD simulation of Mmar_3581 from *Mycobacterium marinum*. Values represent the fraction of simulation time during which each residue adopted PPII backbone dihedral angles, starting from the corresponding AlphaFold Protein Structure Database model (AF‐B2HL21‐F1‐v6). Glycine residues are highlighted in yellow. Results from two additional 1 μs MD simulations are shown in Figures S20 and S21.
**Figure S23.** Additional CD Experiments. In panels A (PG peptide) and B (RT peptide), the solid blue, green, red and violet solid symbols represent samples 1, 2, 3 and 4, respectively, at 5.0°C and the blue, green, red and violet open symbols represent samples 1, 2, 3 and 4 at 65°C. The difference spectra calculated by subtracting the spectra obtained at 65°C from that measured at 5°C are shown in panels C (PG peptide) and D (RT peptide). Related to Figure 6 E&F in the main text.
**Table S1.** PPP results for loop versus PPII helix segments of PGRS33.
**Table S2.** Summary of CD results*.
**Table S3.** Additional benchmarking of PPP.
**Table S4.** NMR spectral parameters.

## Data Availability

Raw NMR data have been deposited in the Biological Magnetic Resonance Data Bank (BMRB) at https://bmrb.io, reference number 53707 and 53708, and the raw CD spectra and MD simulations data have not been deposited in a public repository; all these datasets are available from the corresponding author upon request.
