# Supplementary material for: PolyProline Predictor: A web server for empirical sequence‐based prediction of polyproline II helices
Source: Protein Sci. 2026 Jun 7;35(7):e70675. doi: 10.1002/pro.70675 (PMC13243192; doi:10.1002/pro.70675)
Supplement: Supplementary file 1 — Figure S1. AlphaFold Server structural model of a bacteriophage glycine‐rich protein linked to a polyglycine peptide. (A) Designed variant of a PPII helical bundle domain from the Salmonella phage tail spike protein bound noncovalently to a polyglycine peptide on the left. The high and moderately high confidence of these predictions are colored blue and cyan, respectively, according to the AlphaFold pIDDT score. (B) Alternative perspective of A. In the sequence shown below the structural models, glycine residues are highlighted in yellow. Figure S2. Residue‐resolved PPII populations in the 1‐μs MD simulation of the kidney bean cell wall glycine‐rich protein. Values represent the fraction of simulation time during which each residue adopted PPII backbone dihedral angles, starting from the corresponding AlphaFold Protein Structure Database model (AF‐P10496‐F1‐v6). Glycine residues are highlighted in yellow. Results from two additional 1 μs MD simulations are shown in Figures S3 and S4. Figure S3. Residue‐resolved PPII populations in the second 1‐μs MD simulation of the kidney bean cell wall glycine‐rich protein. Values represent the fraction of simulation time during which each residue adopted PPII backbone dihedral angles, starting from the corresponding AlphaFold Protein Structure Database model (AF‐P10496‐F1‐v6). Glycine residues are highlighted in yellow. Results from two additional 1 μs MD simulations are shown in Figures S2 and S4. Figure S4. Residue‐resolved PPII populations in the third 1‐μs MD simulation of the kidney bean cell wall glycine‐rich protein. Values represent the fraction of simulation time during which each residue adopted PPII backbone dihedral angles, starting from the corresponding AlphaFold Protein Structure Database model (AF‐P10496‐F1‐v6). Glycine residues are highlighted in yellow. Results from two additional 1 μs MD simulations are shown in Figures S2 and S3. Figure S5. Residue‐resolved PPII populations in the 1‐μs MD simulation of the sc [file PRO-35-e70675-s001.docx]

**Supporting Material**


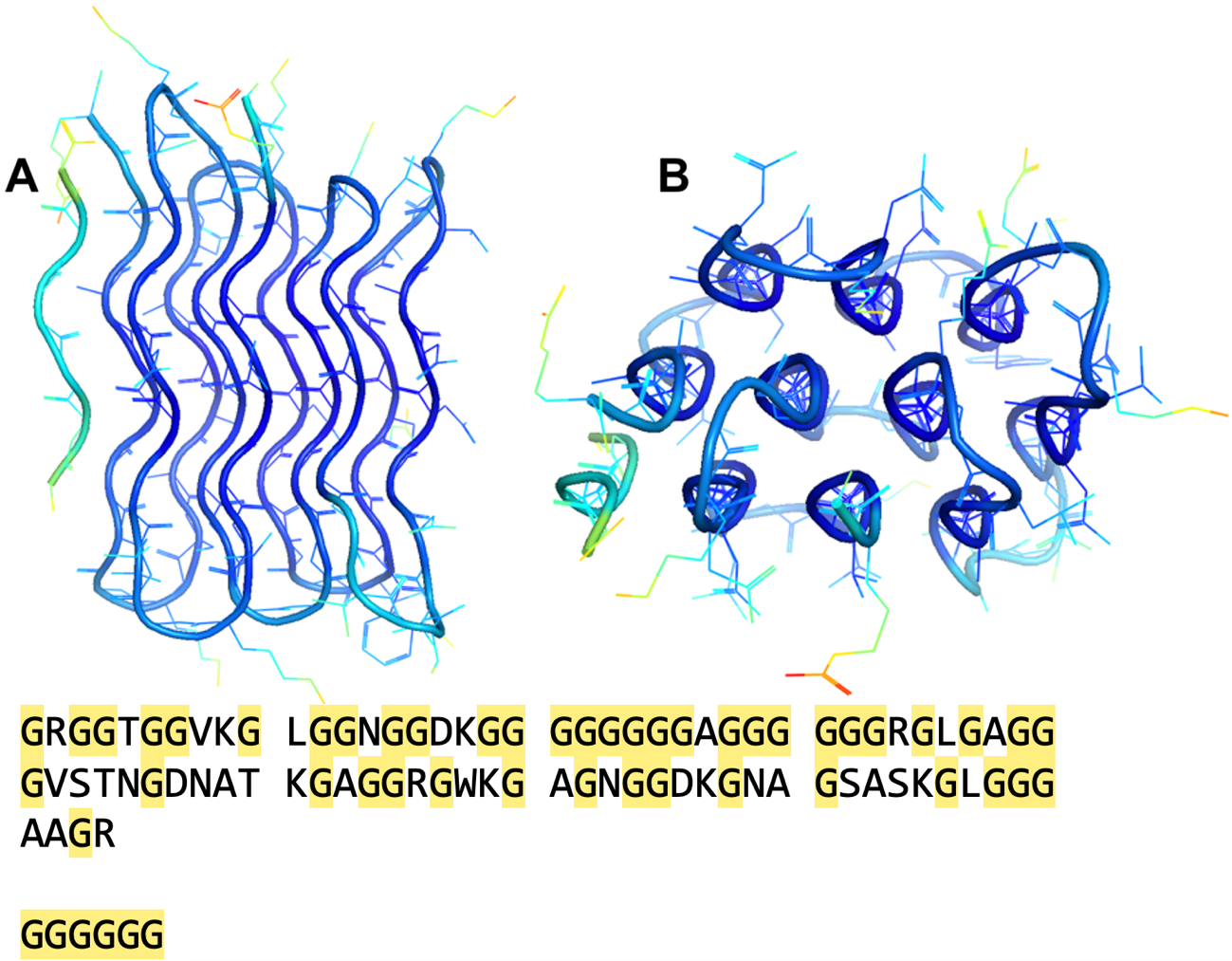


**Figure S1. AlphaFold Server structural model~~s~~ of a bacteriophage glycine-rich protein linked to a polyglycine peptide.** **A**) Designed variant of a PPII helical bundle domain from the Salmonella phage tail spike protein bound noncovalently to a polyglycine peptide on the left. The high and moderately high confidence of these predictions are colored blue and cyan, respectively, according to the AlphaFold pIDDT score. **B**) Alternative perspective of **A**. In the sequence shown below the structural models, glycine residues are highlighted in yellow.


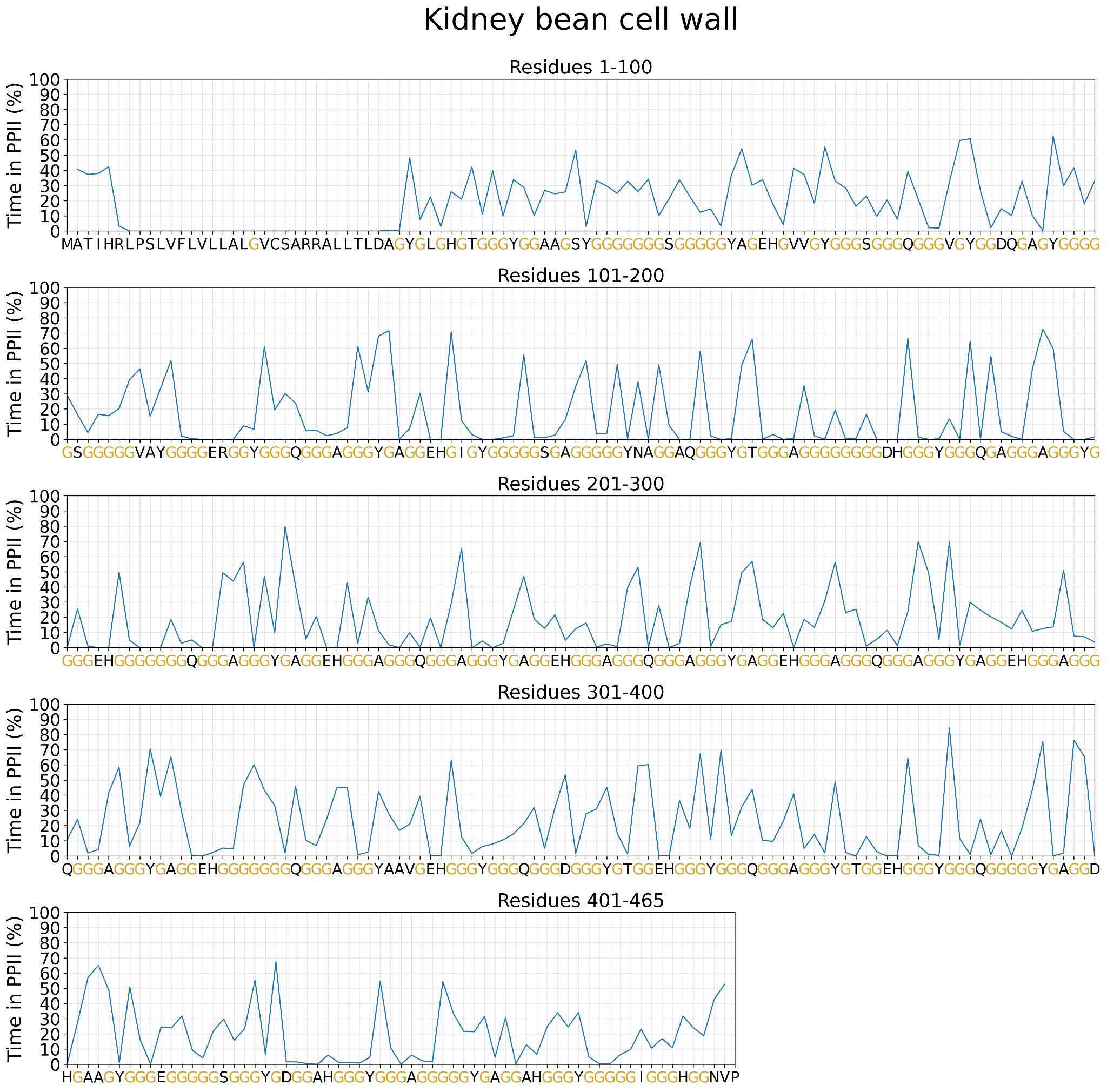


**Figure S2. Residue-resolved PPII populations in the 1-μs MD simulation of the kidney bean cell wall glycine-rich protein.** Values represent the fraction of simulation time during which each residue adopted PPII backbone dihedral angles, starting from the corresponding AlphaFold Protein Structure Database model (AF-P10496-F1-v6). Glycine residues are highlighted in yellow. Results from two additional 1 μs MD simulations are shown in **Figures S3 and S4**.


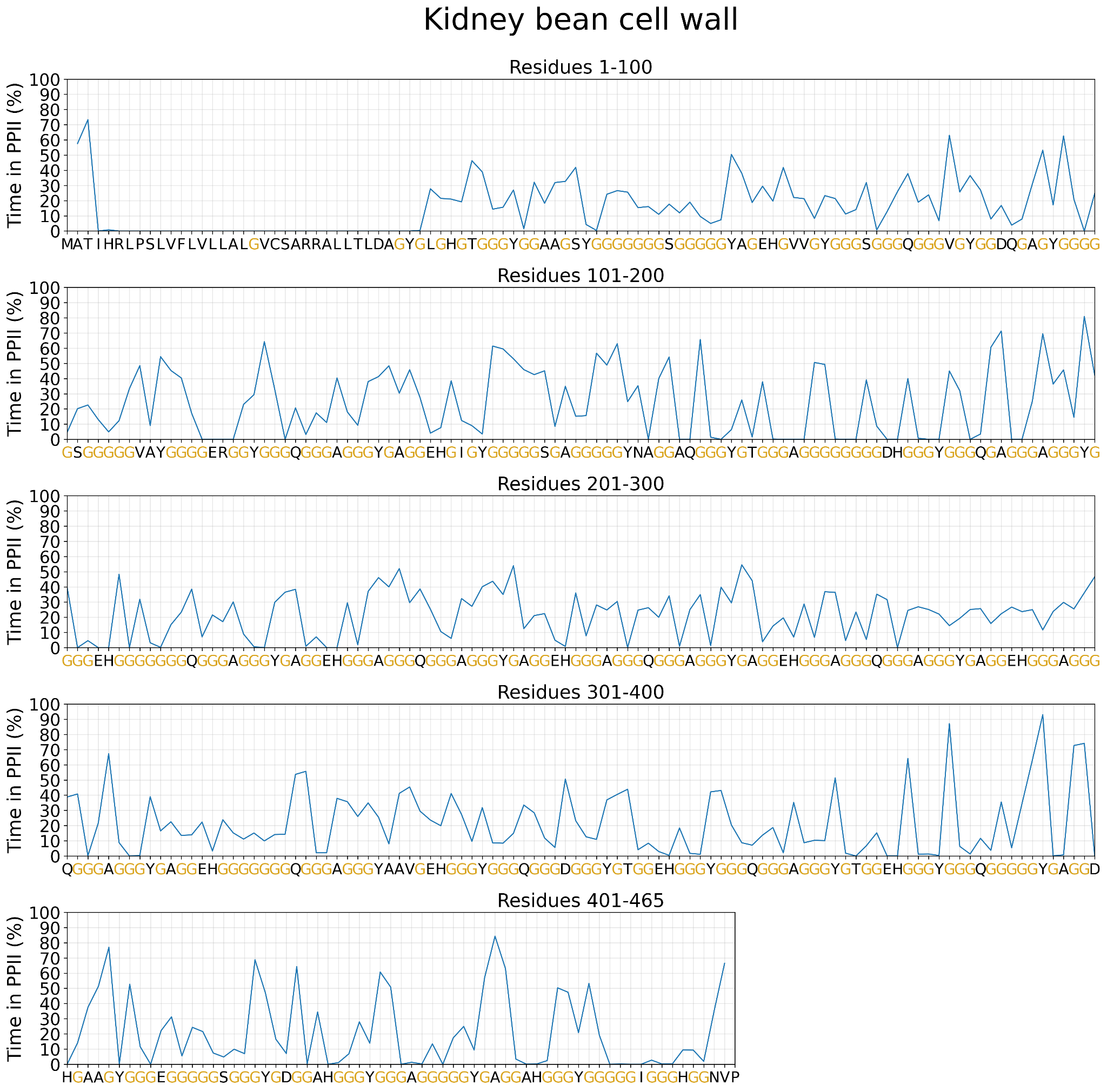


**Figure S3. Residue-resolved PPII populations in the second 1-μs MD simulation of the kidney bean cell wall glycine-rich protein.** Values represent the fraction of simulation time during which each residue adopted PPII backbone dihedral angles, starting from the corresponding AlphaFold Protein Structure Database model (AF-P10496-F1-v6). Glycine residues are highlighted in yellow. Results from two additional 1 μs MD simulations are shown in **Figures S2 and S4**.


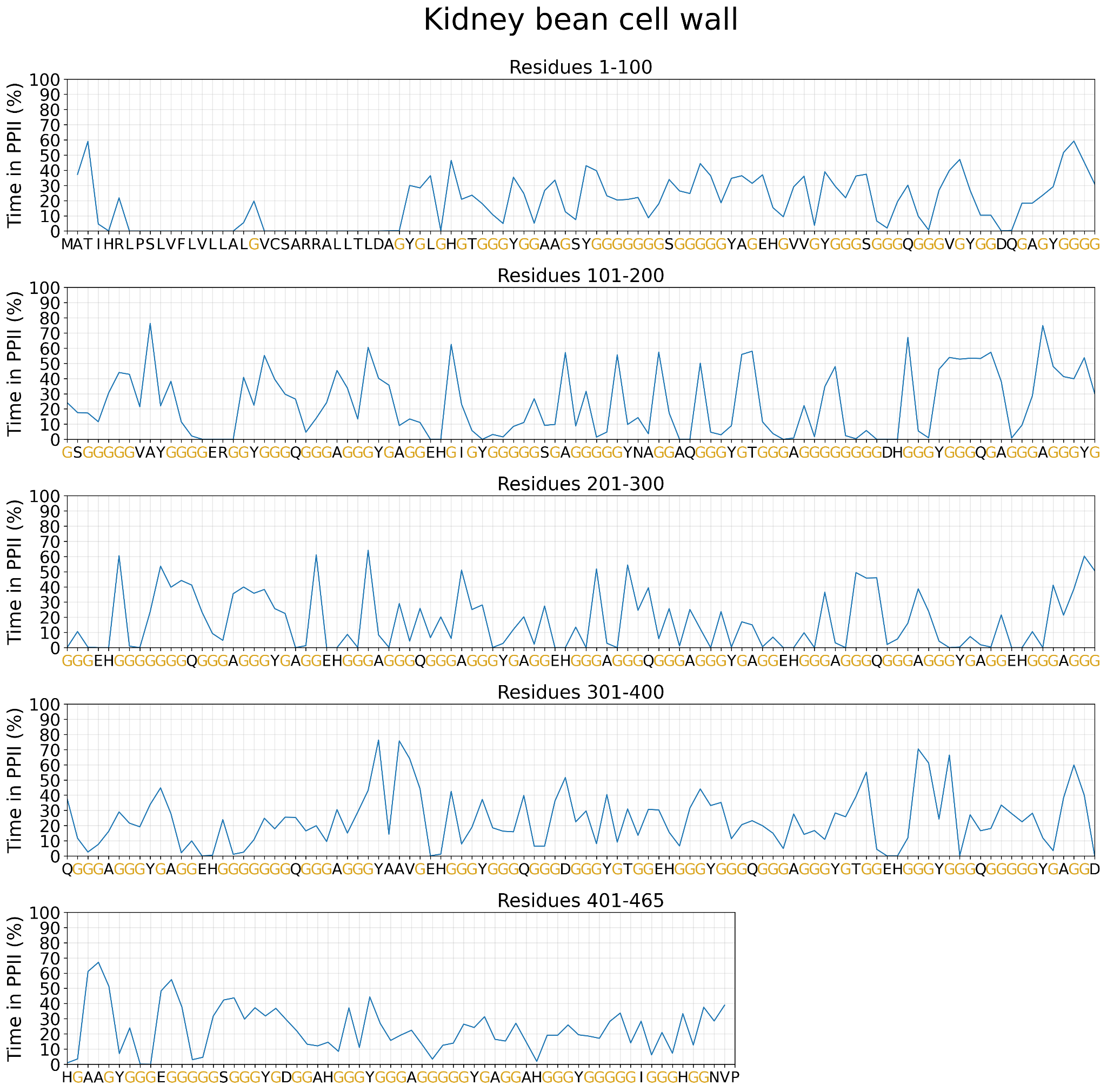


**Figure S4. Residue-resolved PPII populations in the third 1-μs MD simulation of the kidney bean cell wall glycine-rich protein.** Values represent the fraction of simulation time during which each residue adopted PPII backbone dihedral angles, starting from the corresponding AlphaFold Protein Structure Database model (AF-P10496-F1-v6). Glycine residues are highlighted in yellow. Results from two additional 1 μs MD simulations are shown in **Figures S2 and S3**.


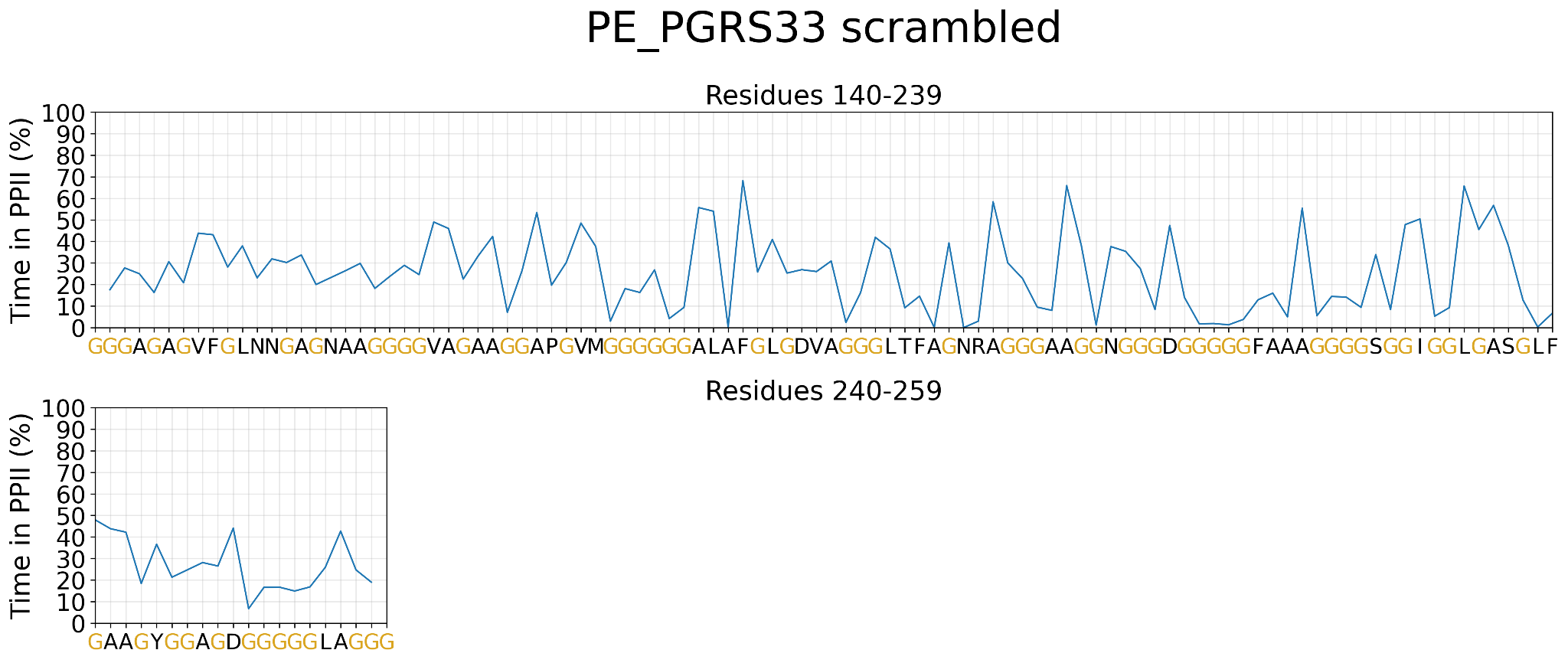


**Figure S5. Residue-resolved PPII populations in the 1-μs MD simulation of the scrambled TRL2 interacting domain of PE_PGRS33 from *Mycobacterium tuberculosis*.** Values represent the fraction of simulation time during which each residue adopted PPII backbone dihedral angles, starting from the generated AlphaFold Server model. Glycine residues are highlighted in yellow. Results from two additional 1 μs MD simulations are shown in **Figures S6 and S7**.


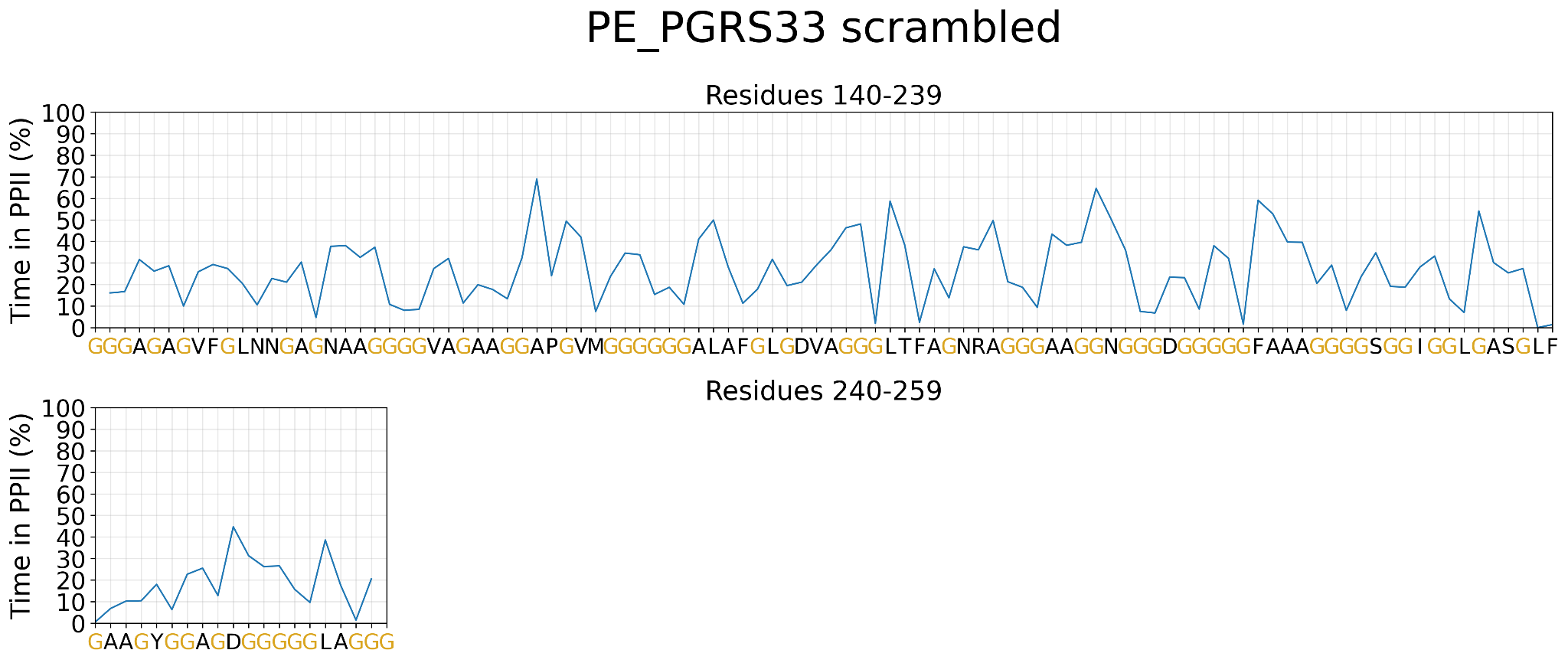


**Figure S6. Residue-resolved PPII populations in the second 1-μs MD simulation of the scrambled TRL2 interacting domain of PE_PGRS33 from *Mycobacterium tuberculosis*.** Values represent the fraction of simulation time during which each residue adopted PPII backbone dihedral angles, starting from the generated AlphaFold Server model. Glycine residues are highlighted in yellow. Results from two additional 1 μs MD simulations are shown in **Figures S5 and S7**.


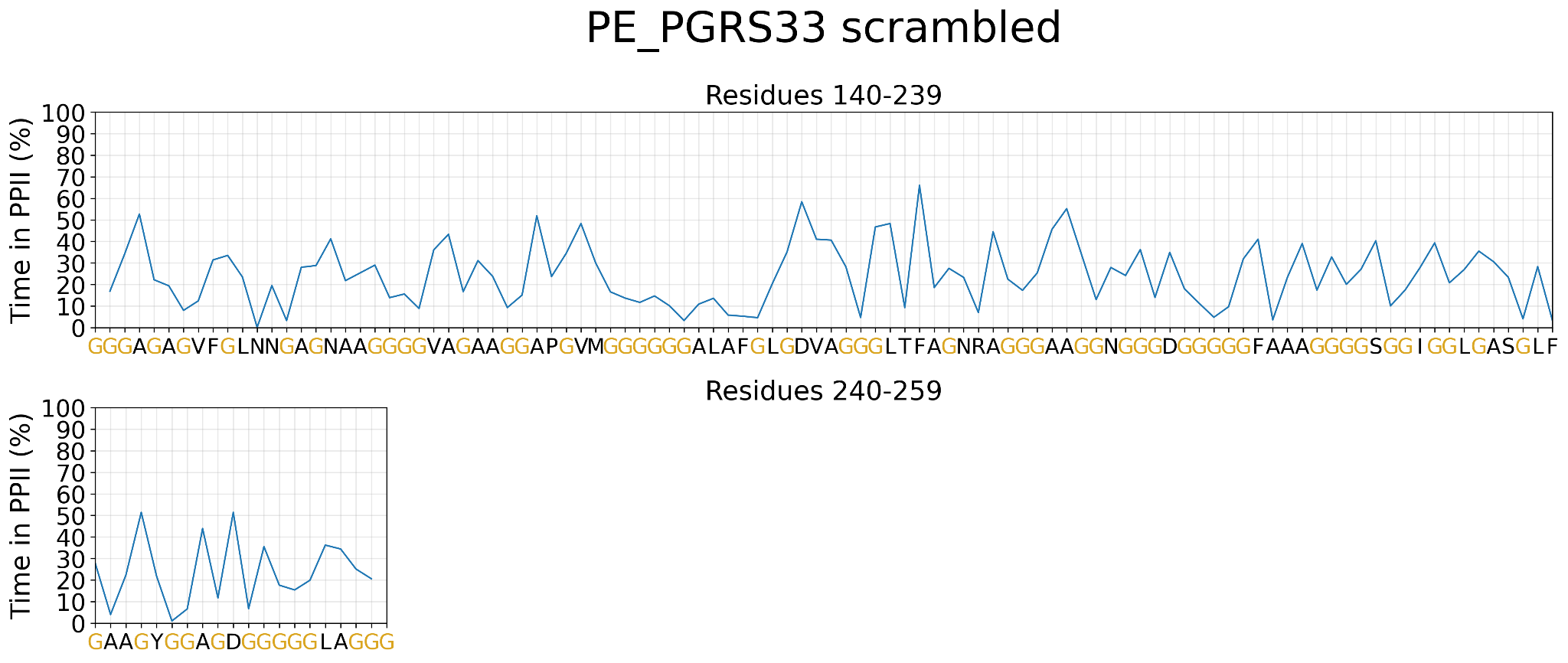


**Figure S7. Residue-resolved PPII populations in the third 1-μs MD simulation of the scrambled TRL2 interacting domain of PE_PGRS33 from *Mycobacterium tuberculosis*.** Values represent the fraction of simulation time during which each residue adopted PPII backbone dihedral angles, starting from the generated AlphaFold Server model. Glycine residues are highlighted in yellow. Results from two additional 1 μs MD simulations are shown in **Figures S5 and S6**.

**
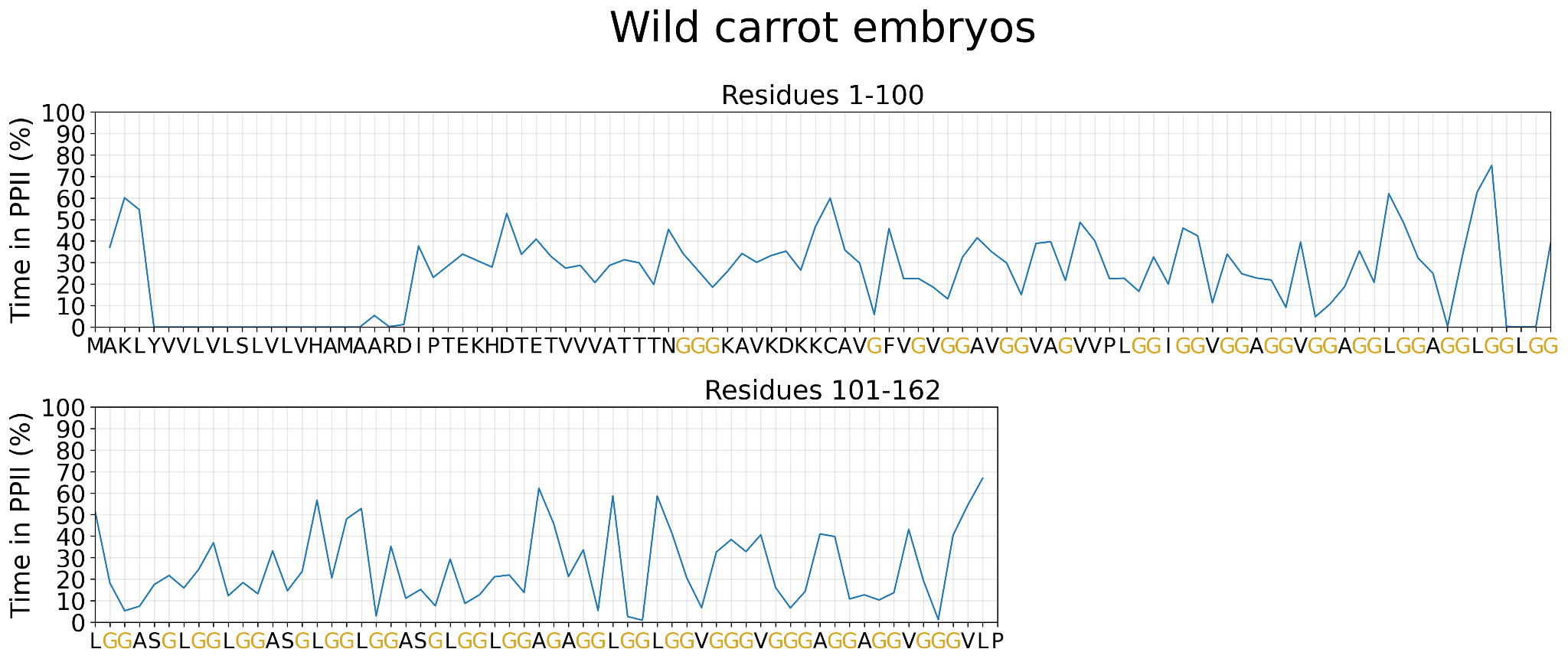
**

**Figure S8. Residue-resolved PPII populations in the 1-μs MD simulation of the wild carrot embryo glycine-rich protein.** Values represent the fraction of simulation time during which each residue adopted PPII backbone dihedral angles, starting from the corresponding AlphaFold Protein Structure Database model (AF-Q39691-F1-v6). Glycine residues are highlighted in yellow. Results from two additional 1 μs MD simulations are shown in **Figures S9 and S10**.


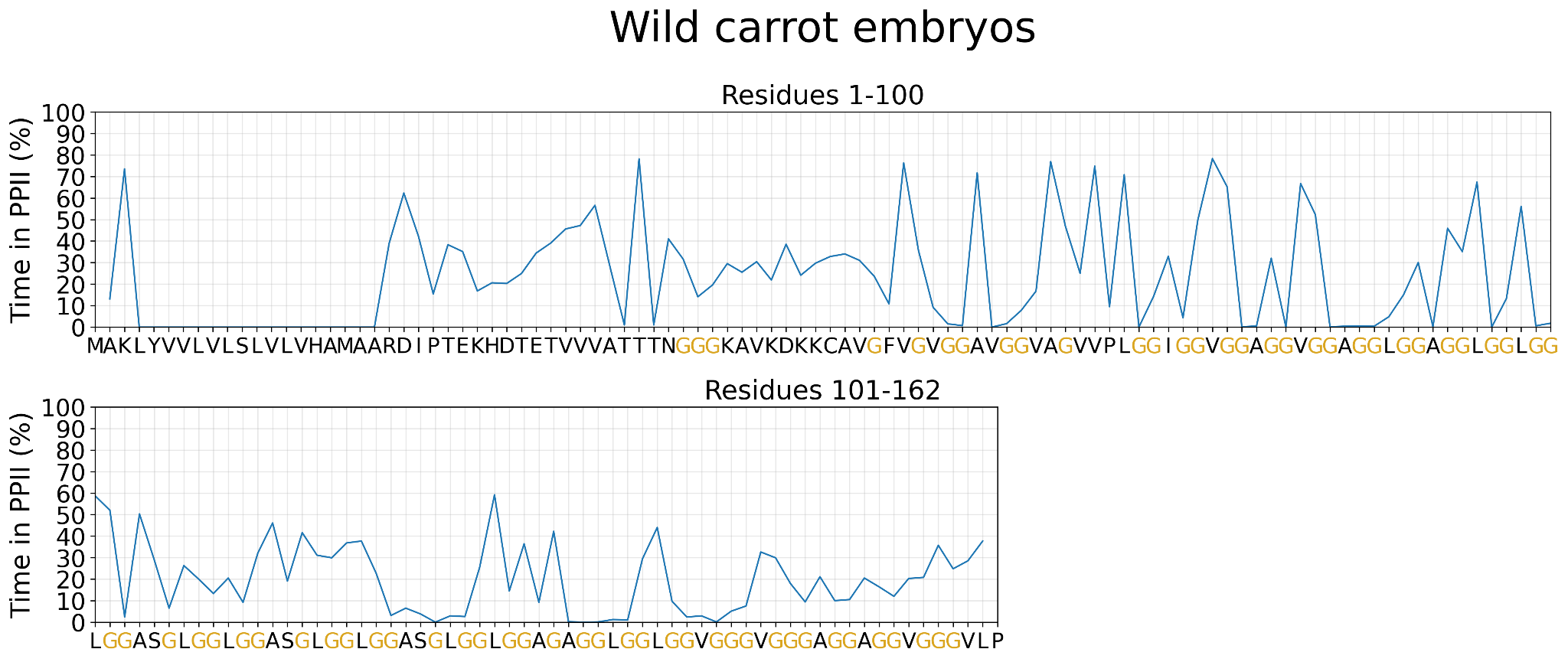


**Figure S9. Residue-resolved PPII populations in the second 1-μs MD simulation of the wild carrot embryo glycine-rich protein.** Values represent the fraction of simulation time during which each residue adopted PPII backbone dihedral angles, starting from the corresponding AlphaFold Protein Structure Database model (AF-Q39691-F1-v6). Glycine residues are highlighted in yellow. Results from two additional 1 μs MD simulations are shown in **Figures S8 and S10**.


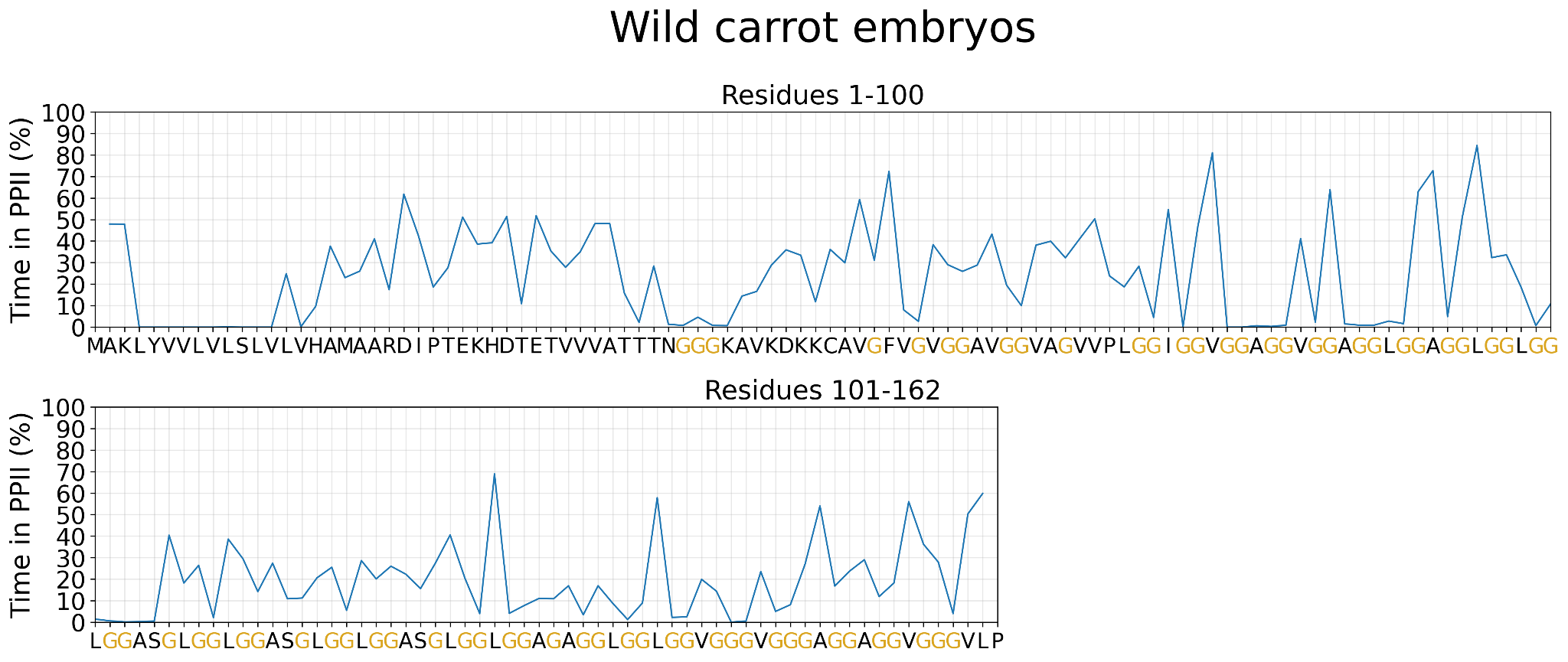


**Figure S10. Residue-resolved PPII populations in the third 1-μs MD simulation of the wild carrot embryo glycine-rich protein.** Values represent the fraction of simulation time during which each residue adopted PPII backbone dihedral angles, starting from the corresponding AlphaFold Protein Structure Database model (AF-Q39691-F1-v6). Glycine residues are highlighted in yellow. Results from two additional 1 μs MD simulations are shown in **Figures S8 and S9**.


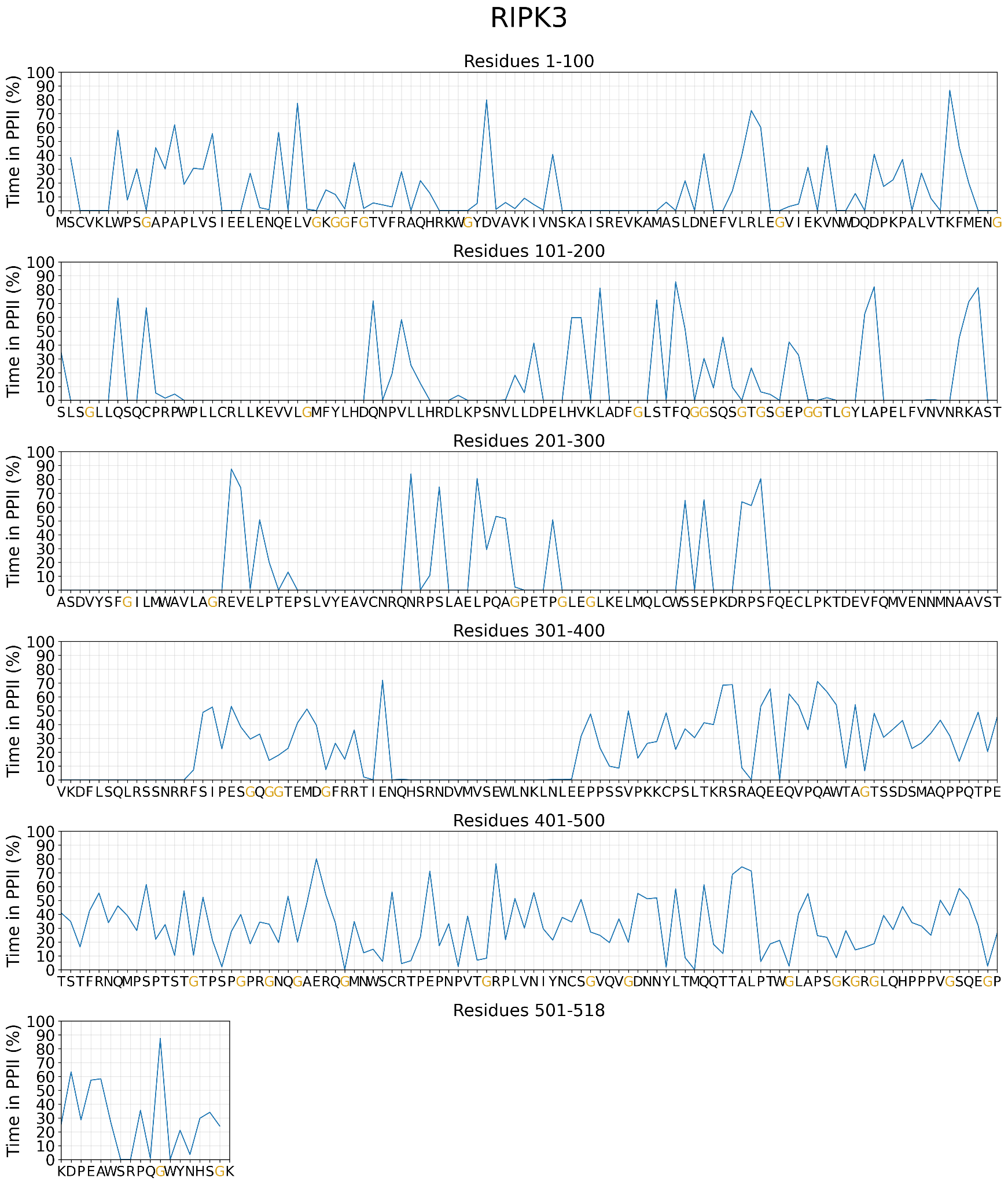


**Figure S11. Residue-resolved PPII populations in the 1-μs MD simulation of human RIPK3.** Values represent the fraction of simulation time during which each residue adopted PPII backbone dihedral angles, starting from the corresponding AlphaFold Protein Structure Database model (AF-Q9Y572-F1-v6). Glycine residues are highlighted in yellow.Results from two additional 1 μs MD simulations are shown in **Figures S12 and S13**.


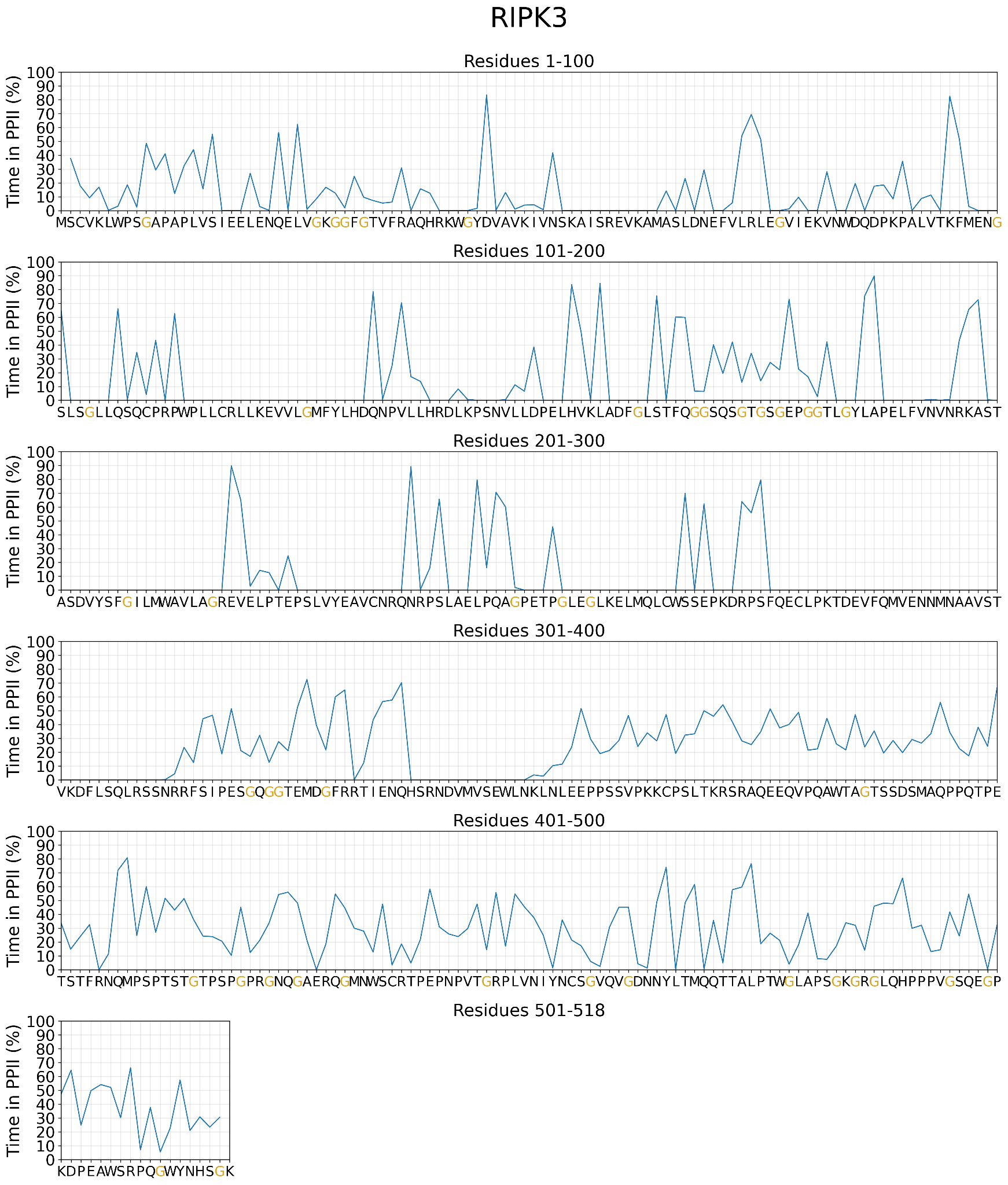


**Figure S12. Residue-resolved PPII populations in the second 1-μs MD simulation of human RIPK3.** Values represent the fraction of simulation time during which each residue adopted PPII backbone dihedral angles, starting from the corresponding AlphaFold Protein Structure Database model (AF-Q9Y572-F1-v6). Glycine residues are highlighted in yellow. Results from two additional 1 μs MD simulations are shown in **Figures S11 and S13.**


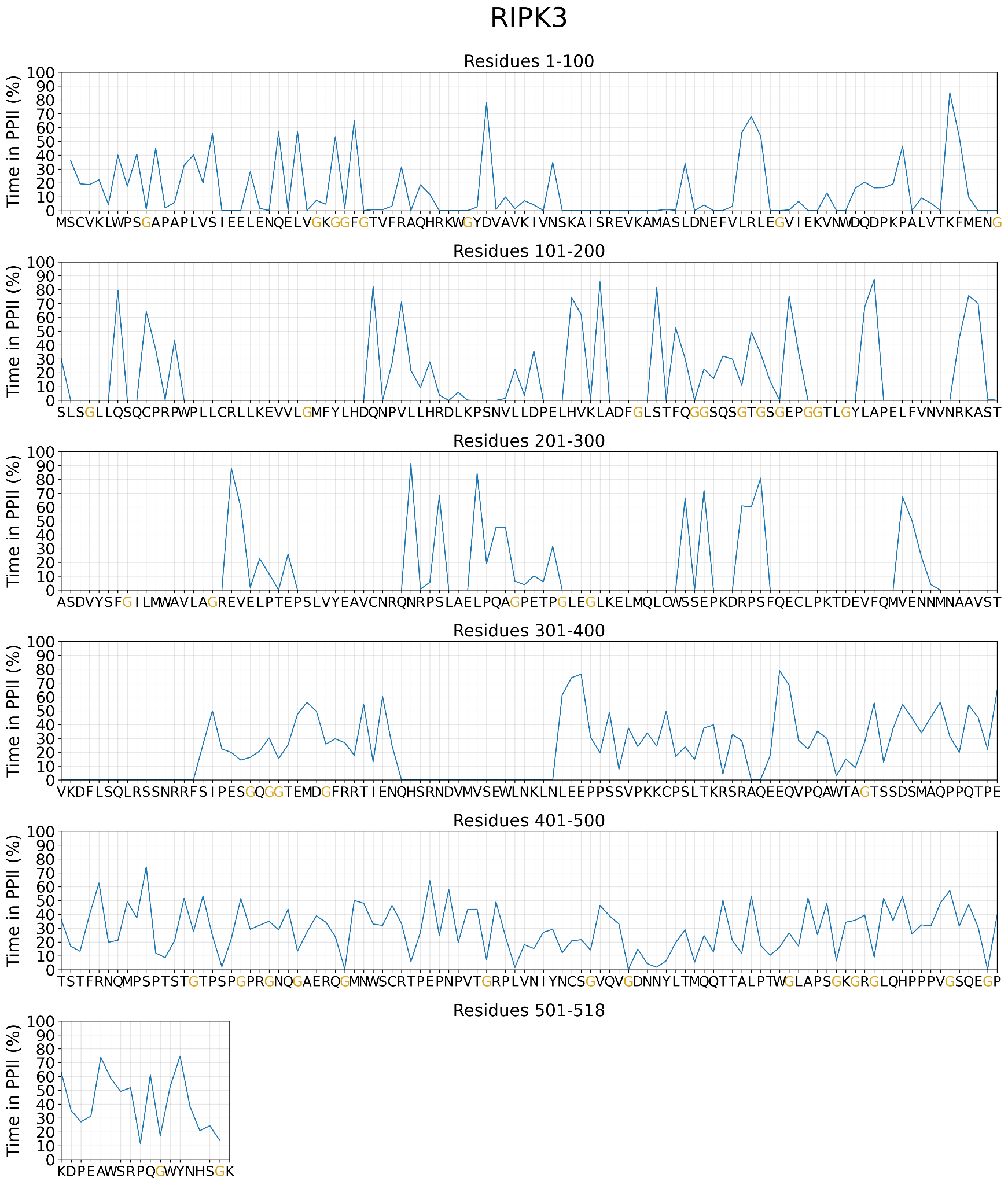


**Figure S13. Residue-resolved PPII populations in the third 1-μs MD simulation of human RIPK3.** Values represent the fraction of simulation time during which each residue adopted PPII backbone dihedral angles, starting from the corresponding AlphaFold Protein Structure Database model (AF-Q9Y572-F1-v6). Glycine residues are highlighted in yellow. Results from two additional 1 μs MD simulations are shown in **Figures S11 and S12**.


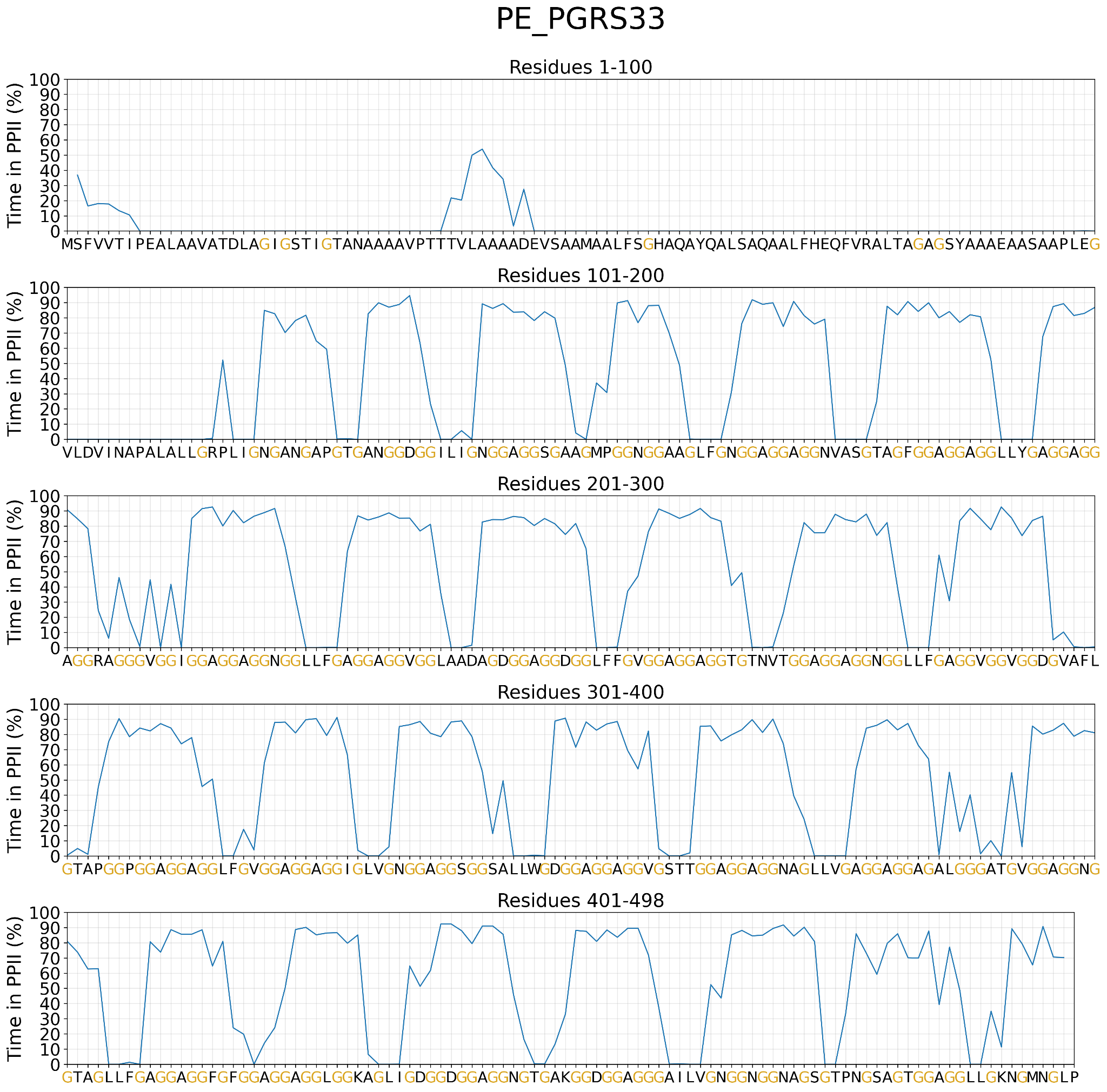


**Figure S14. Residue-resolved PPII populations in the 1-μs MD simulation of PE_PGRS33 from *Mycobacterium tuberculosis*.** Values represent the fraction of simulation time during which each residue adopted PPII backbone dihedral angles, starting from the corresponding AlphaFold Protein Structure Database model (AF-P9WIF5-F1-v6). Glycine residues are highlighted in yellow. Results from two additional 1 μs MD simulations are shown in **Figures S15 and S16**.


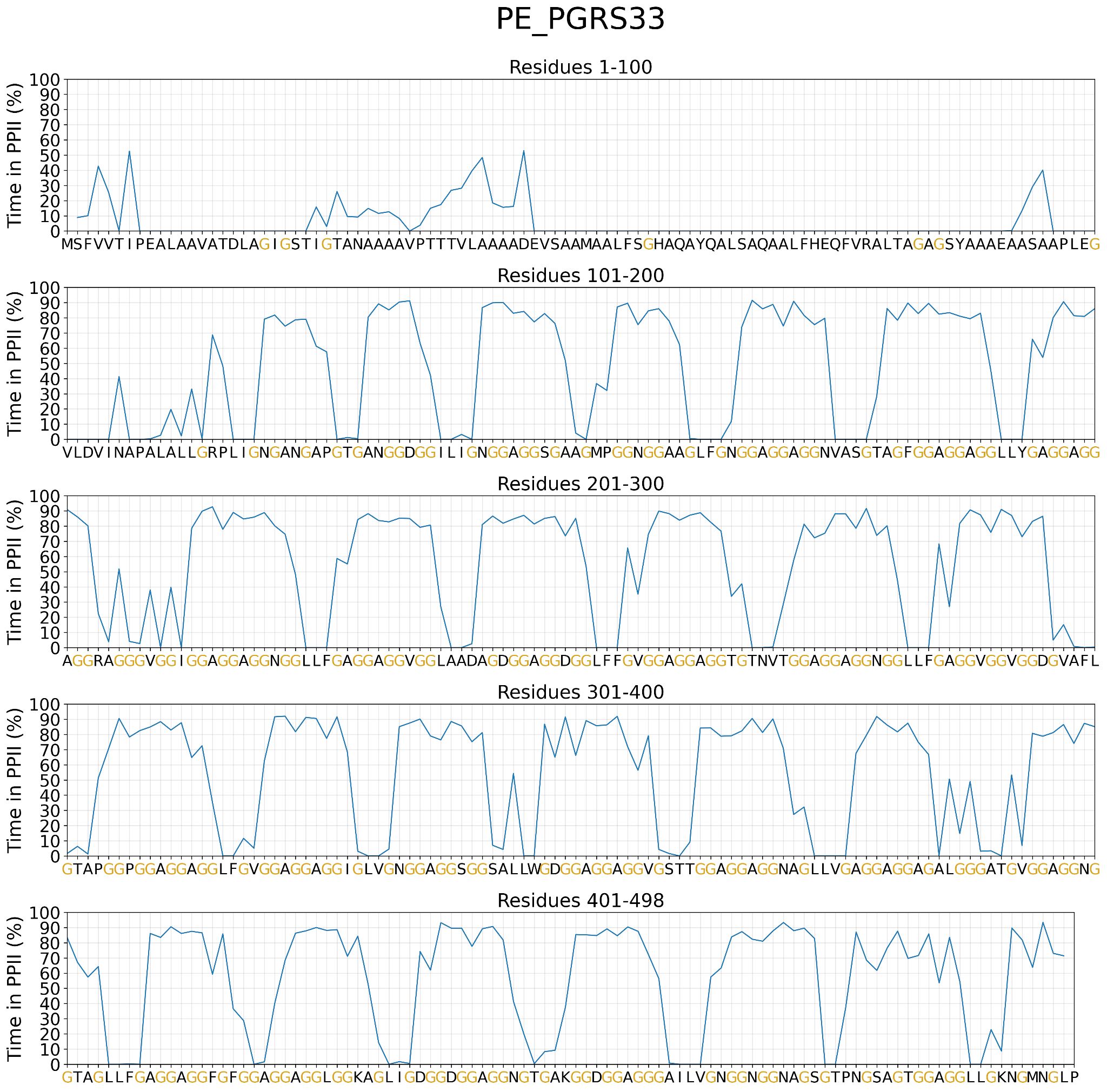


**Figure S15. Residue-resolved PPII populations in the second 1-μs MD simulation of PE_PGRS33 from *Mycobacterium tuberculosis*.** Values represent the fraction of simulation time during which each residue adopted PPII backbone dihedral angles, starting from the corresponding AlphaFold Protein Structure Database model (AF-P9WIF5-F1-v6). Glycine residues are highlighted in yellow. Results from two additional 1 μs MD simulations are shown in **Figures S14 and S16**.


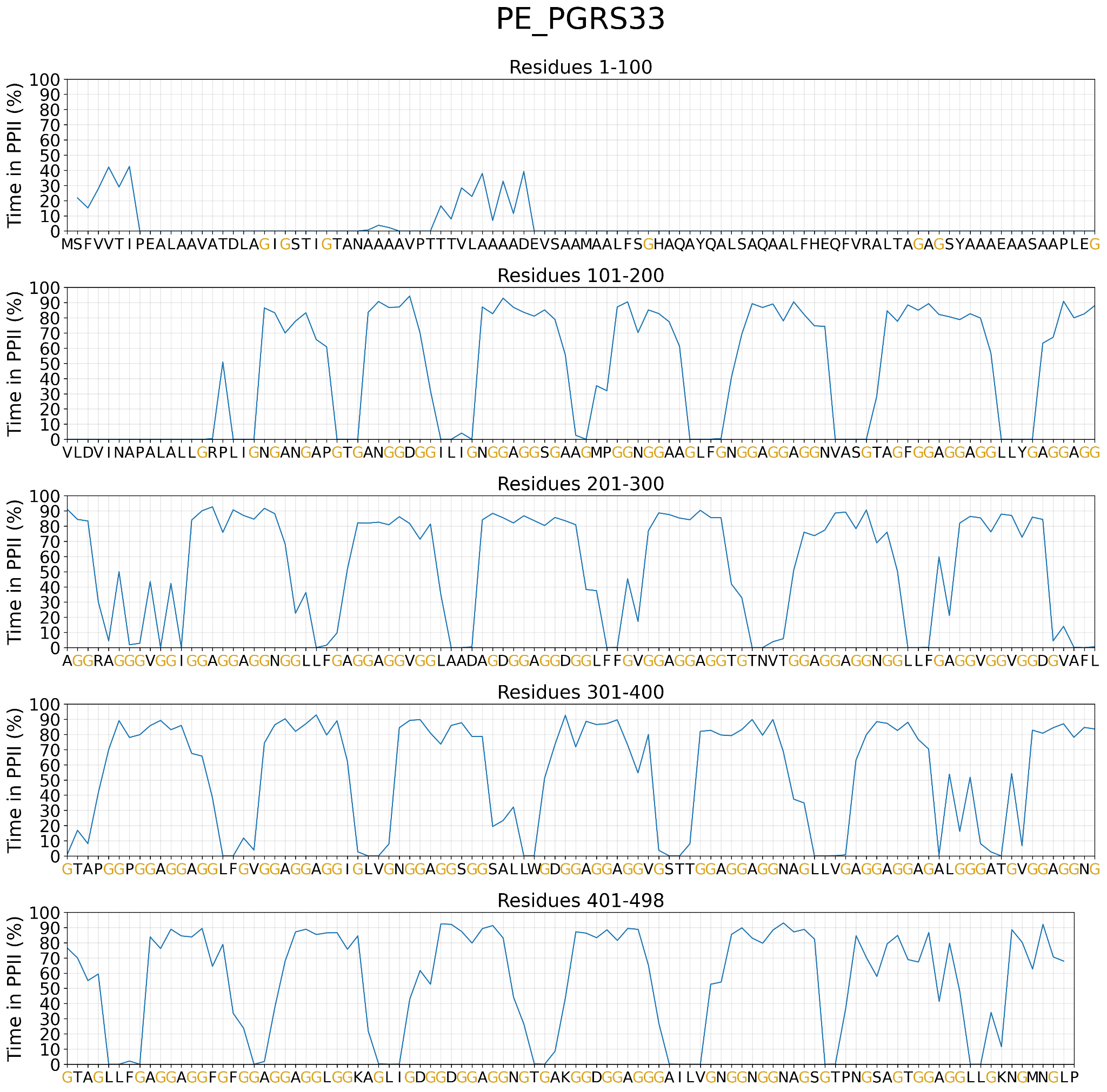


**Figure S16. Residue-resolved PPII populations in the third 1-μs MD simulation of PE_PGRS33 from *Mycobacterium tuberculosis*.** Values represent the fraction of simulation time during which each residue adopted PPII backbone dihedral angles, starting from the corresponding AlphaFold Protein Structure Database model (AF-P9WIF5-F1-v6). Glycine residues are highlighted in yellow. Results from two additional 1 μs MD simulations are shown in **Figures S14 and S15**.


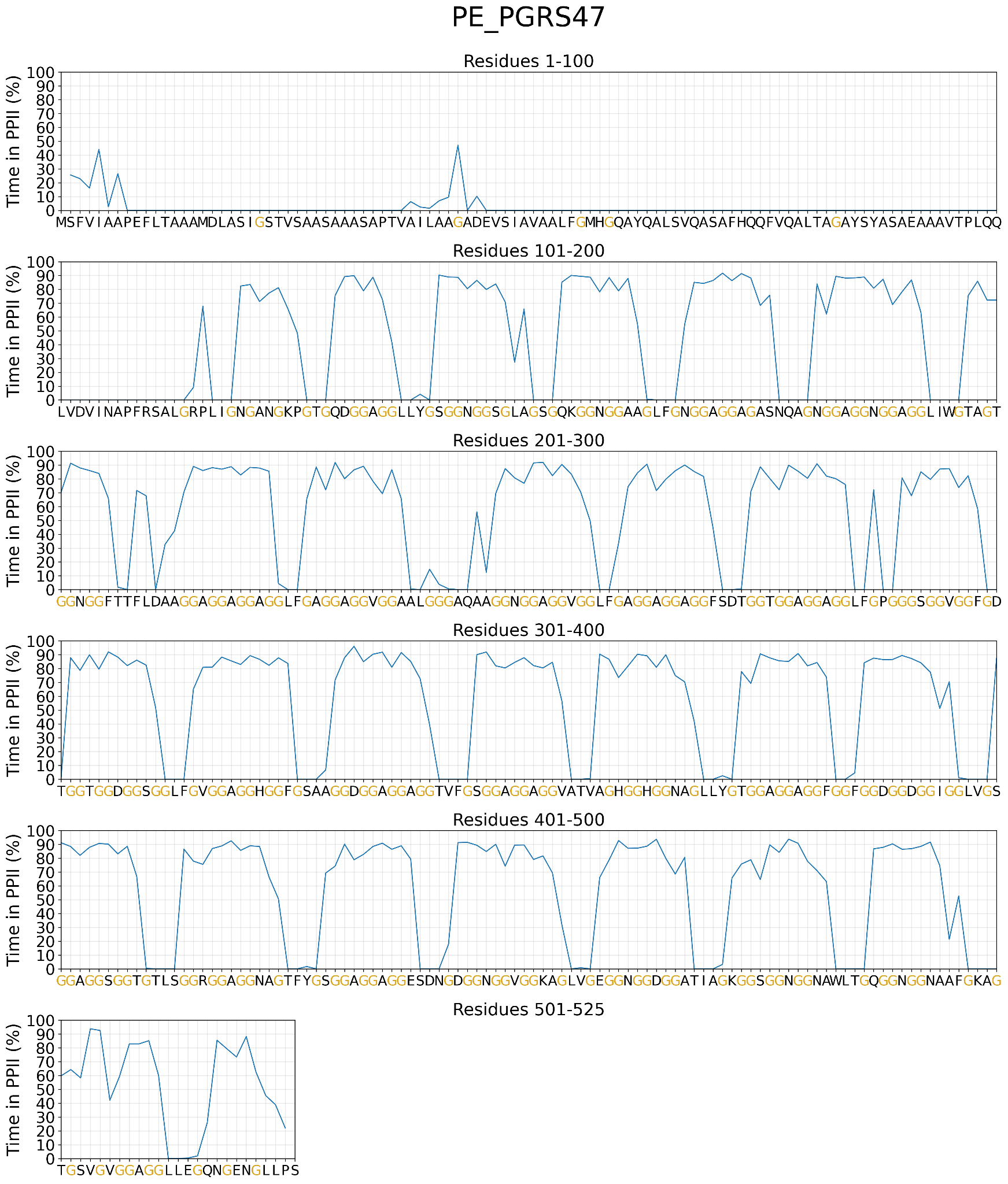


**Figure S17. Residue-resolved PPII populations in the 1-μs MD simulation of PE_PGRS47 from *Mycobacterium tuberculosis*.** Values represent the fraction of simulation time during which each residue adopted PPII backbone dihedral angles, starting from the corresponding AlphaFold Protein Structure Database model (AF-Q79FB3-F1-v6). Glycine residues are highlighted in yellow. Results from two additional 1 μs MD simulations are shown in **Figures S18 and S19**.


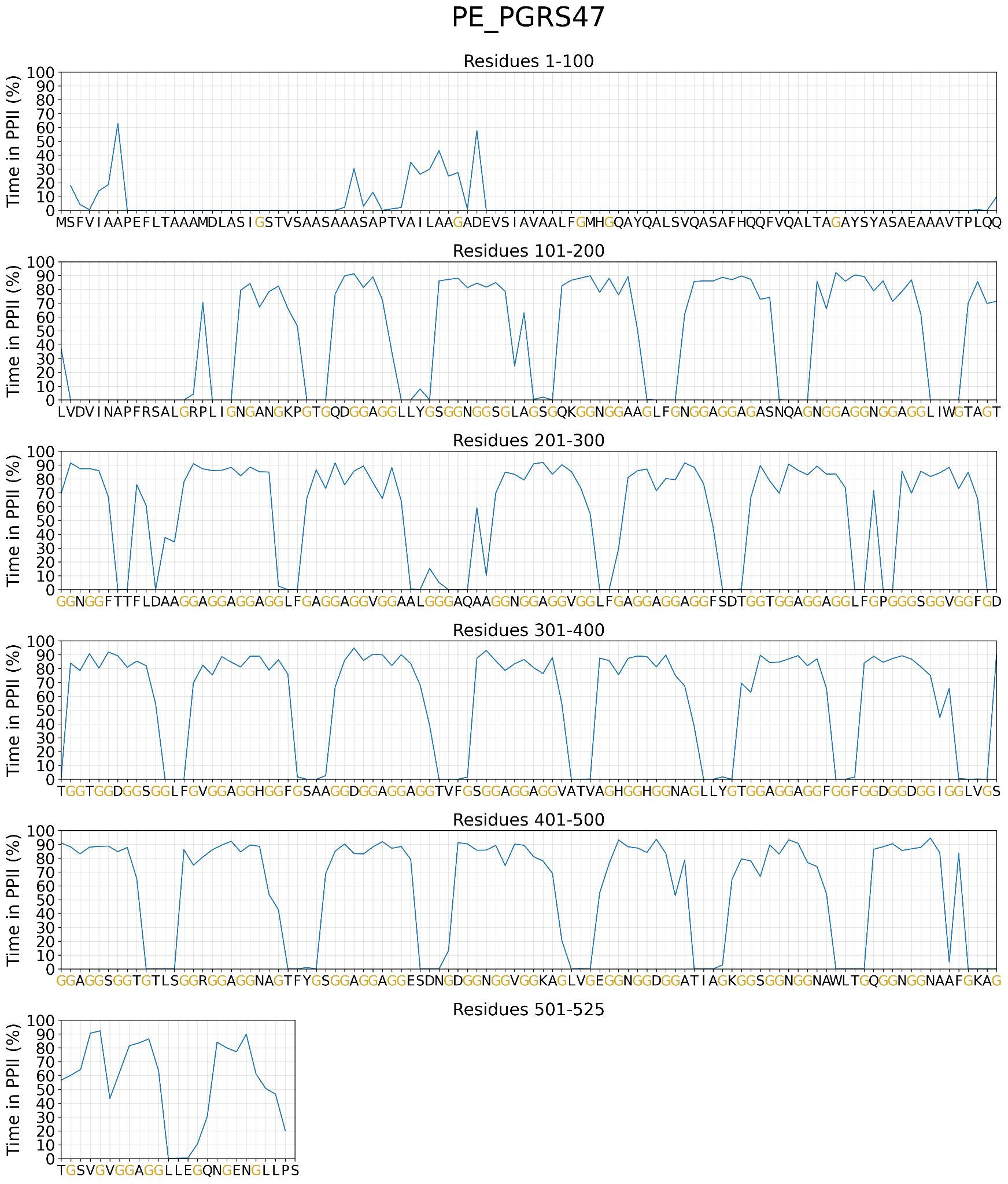


**Figure S18. Residue-resolved PPII populations in the second 1-μs MD simulation of PE_PGRS47 from *Mycobacterium tuberculosis*.** Values represent the fraction of simulation time during which each residue adopted PPII backbone dihedral angles, starting from the corresponding AlphaFold Protein Structure Database model (AF-Q79FB3-F1-v6). Glycine residues are highlighted in yellow. Results from two additional 1 μs MD simulations are shown in **Figures S17 and S19**.


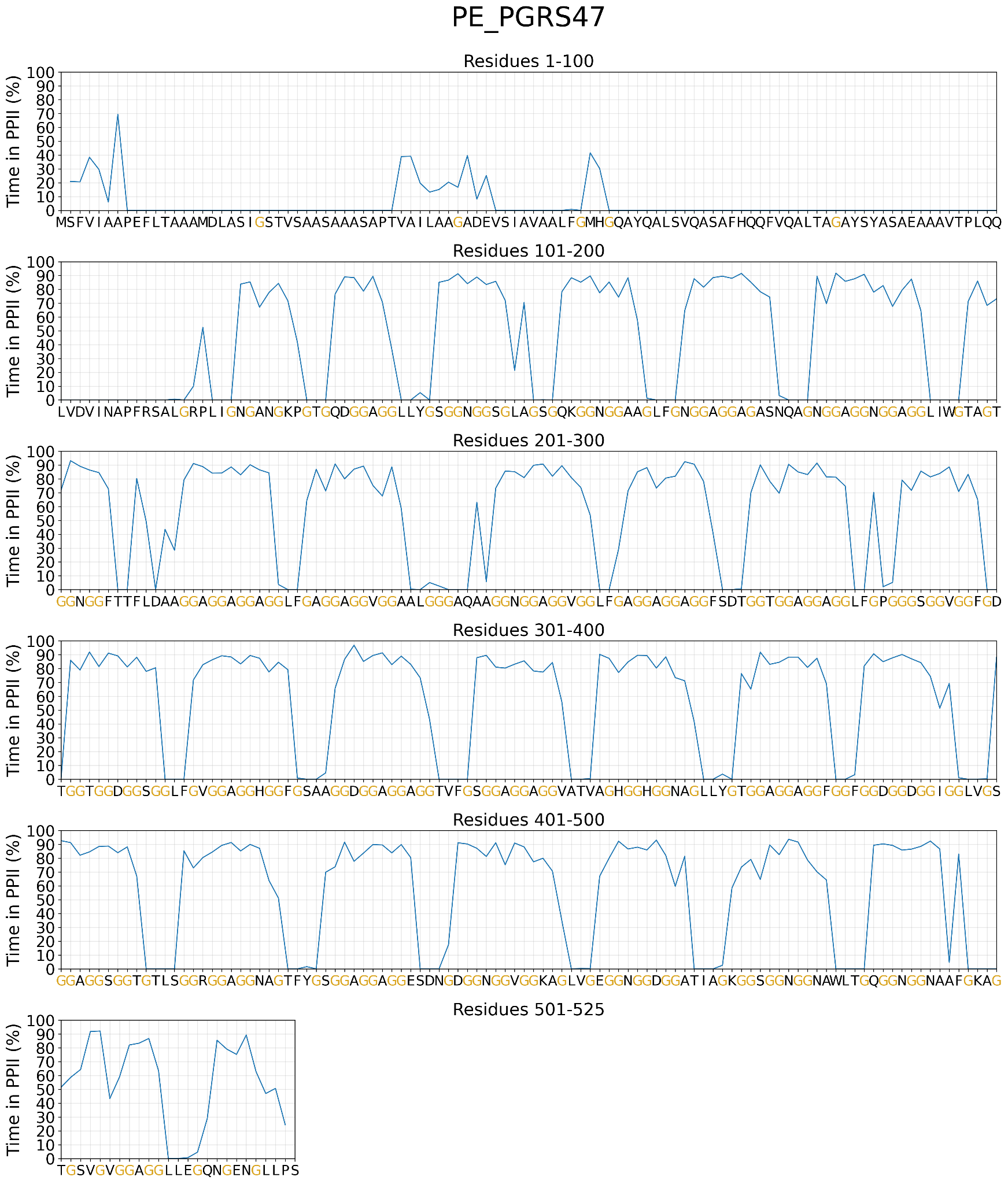


**Figure S19. Residue-resolved PPII populations in the third 1-μs MD simulation of PE_PGRS47 from *Mycobacterium tuberculosis*.** Values represent the fraction of simulation time during which each residue adopted PPII backbone dihedral angles, starting from the corresponding AlphaFold Protein Structure Database model (AF-Q79FB3-F1-v6). Glycine residues are highlighted in yellow. Results from two additional 1 μs MD simulations are shown in **Figures S17 and S18**.


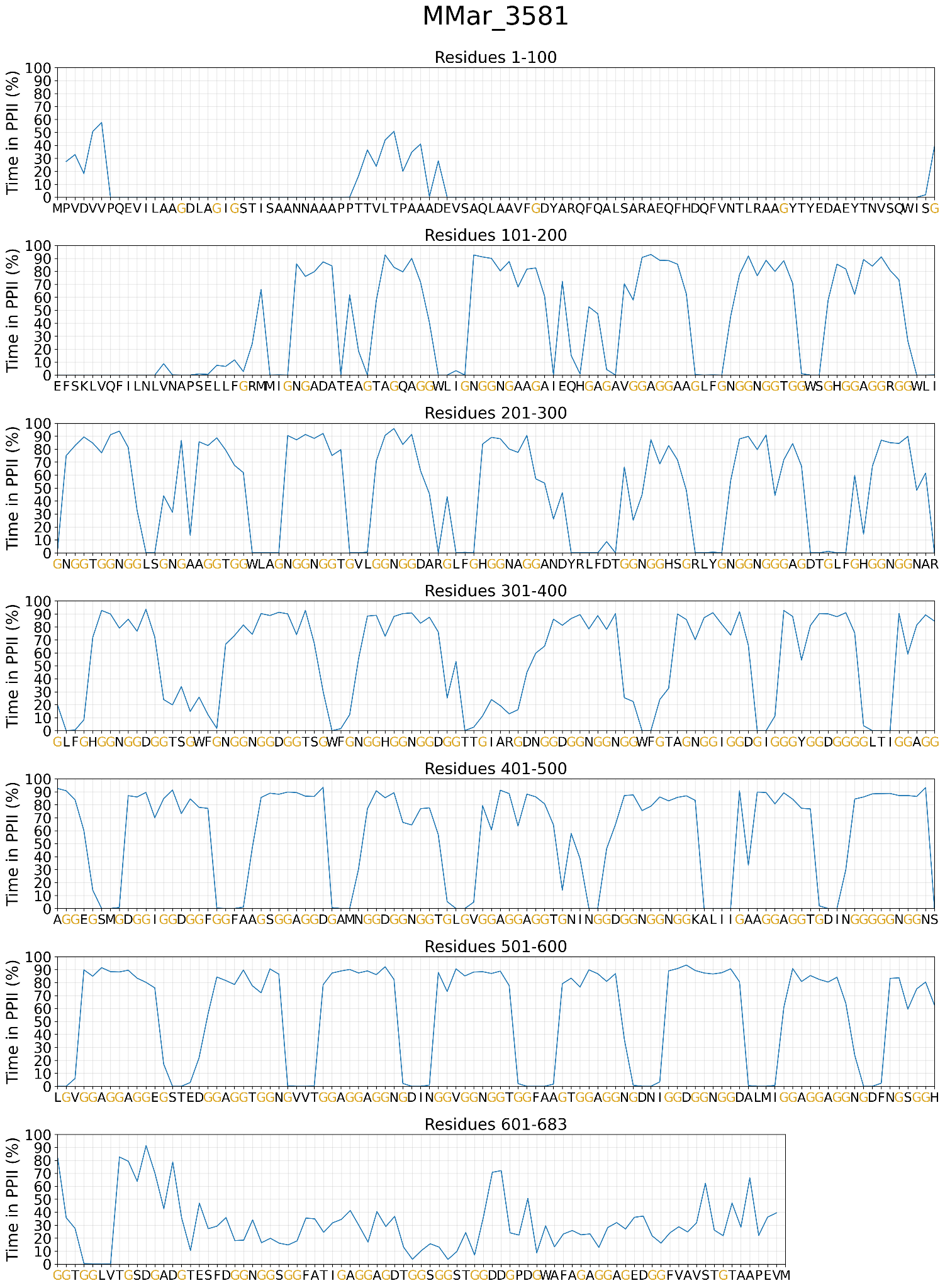


**Figure S20. Residue-resolved PPII populations in the 1-μs MD simulation of Mmar_3581 from *Mycobacterium marinum*.** Values represent the fraction of simulation time during which each residue adopted PPII backbone dihedral angles, starting from the corresponding AlphaFold Protein Structure Database model (AF-B2HL21-F1-v6). Glycine residues are highlighted in yellow. Results from two additional 1 μs MD simulations are shown in **Figures S21 and S22**.


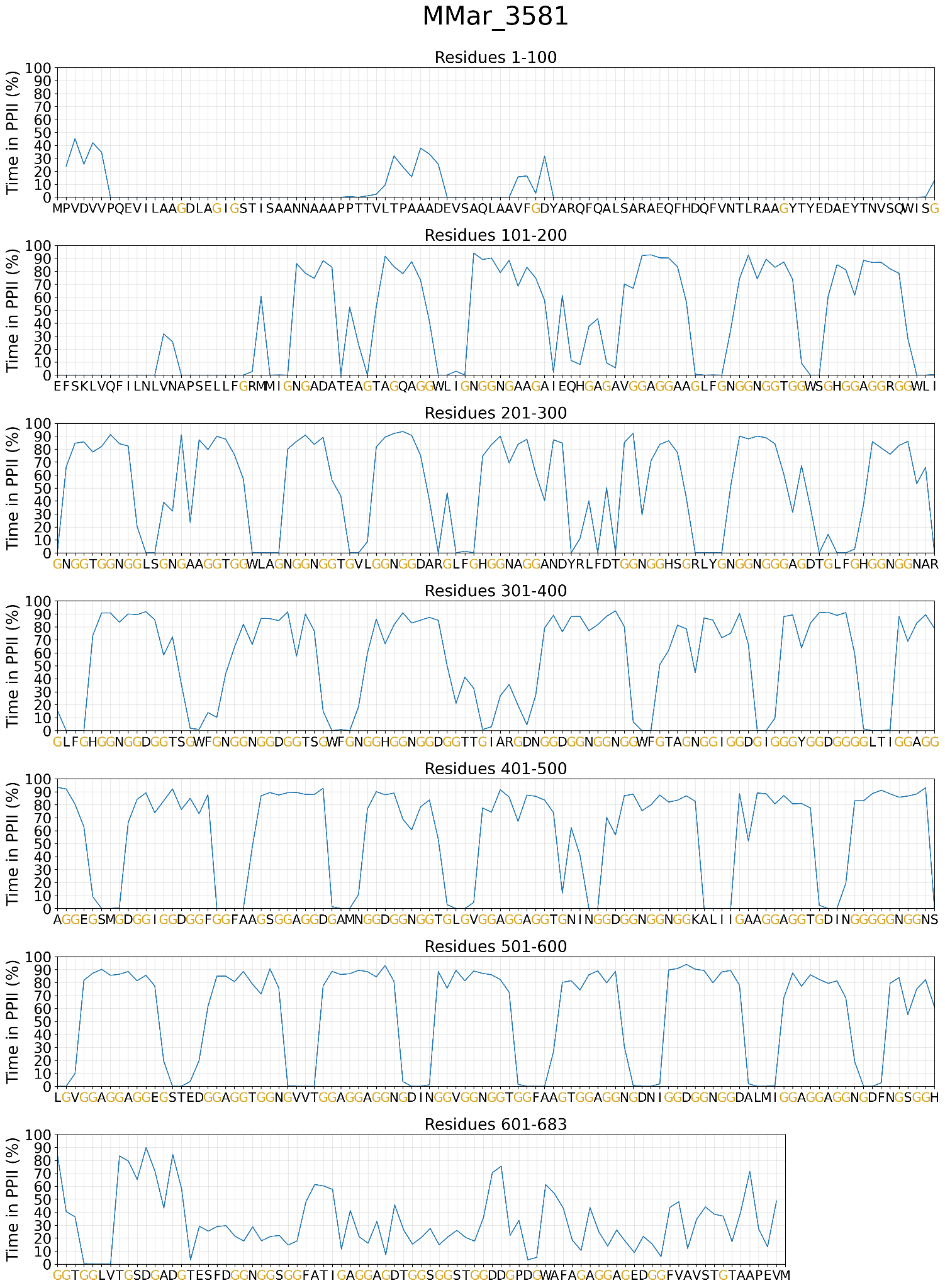


**Figure S21. Residue-resolved PPII populations in the second 1-μs MD simulation of Mmar_3581 from *Mycobacterium marinum*.** Values represent the fraction of simulation time during which each residue adopted PPII backbone dihedral angles, starting from the corresponding AlphaFold Protein Structure Database model (AF-B2HL21-F1-v6). Glycine residues are highlighted in yellow. Results from two additional 1 μs MD simulations are shown in **Figures S20 and S22**.


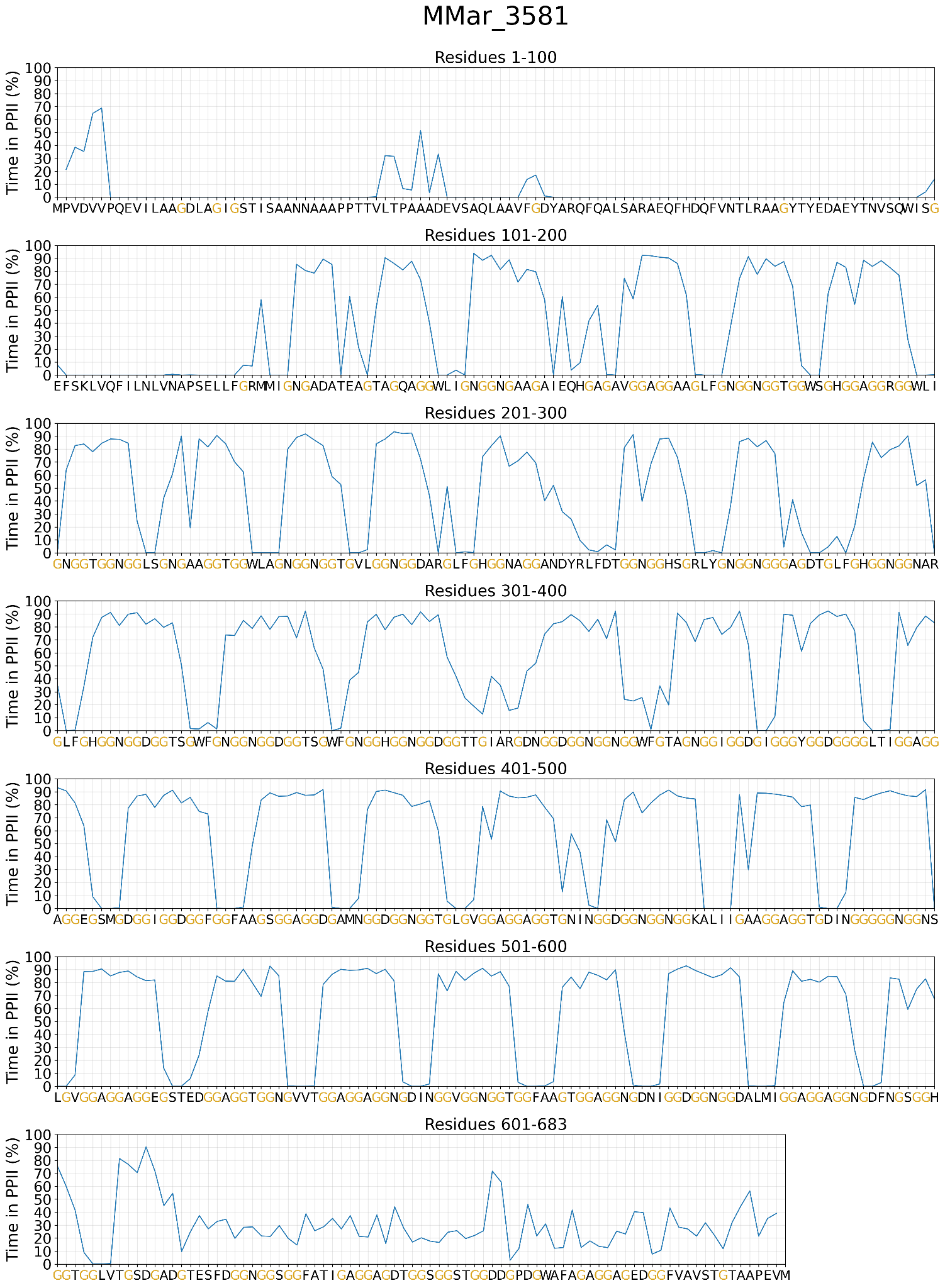


**Figure S22. Residue-resolved PPII populations in the third 1-μs MD simulation of Mmar_3581 from *Mycobacterium marinum*.** Values represent the fraction of simulation time during which each residue adopted PPII backbone dihedral angles, starting from the corresponding AlphaFold Protein Structure Database model (AF-B2HL21-F1-v6). Glycine residues are highlighted in yellow. Results from two additional 1 μs MD simulations are shown in **Figures S20 and S21**.


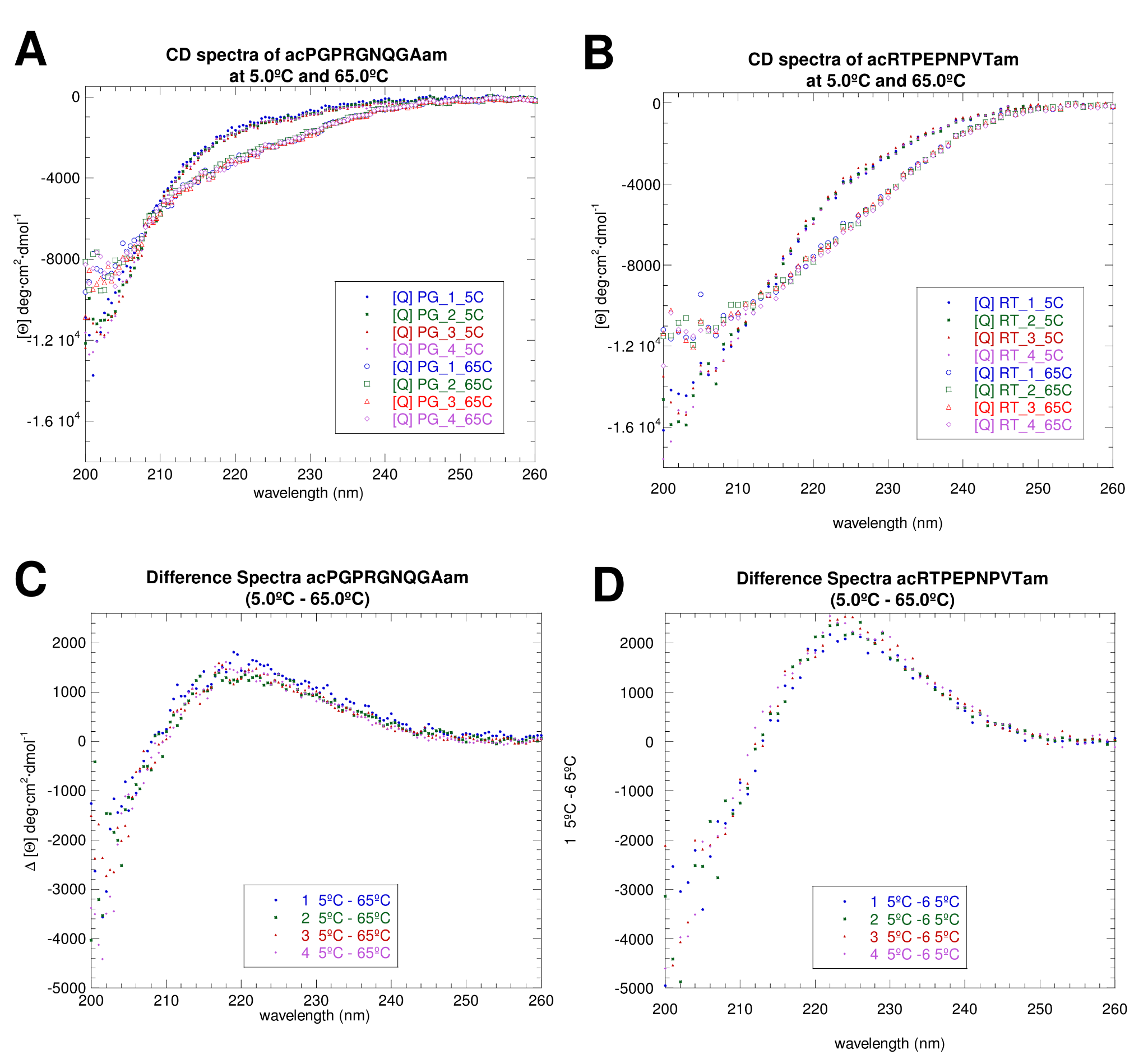


**Figure S23. Additional CD Experiments.** In panels **A (PG peptide)** and **B (RT peptide)**, the solid blue, green, red and violet solid symbols represent samples 1, 2, 3 and 4, respectively, at 5.0ºC and the blue, green, red and violet open symbols represent samples 1, 2, 3 and 4 at 65ºC. The difference spectra calculated by subtracting the spectra obtained at 65ºC from that measured at 5ºC are shown in panels **C (PG peptide)** and **D (RT peptide)**. Related to **Figure 6 E&F** in the main text.

**Table S1.** PPP results for loop versus PPII helix segments of PGRS33

| PGRS33  Hydrophobic loop segment  (< 20% PPII by MD) | PPP Number of protein “hits”* | PGRS33  Polar loop  segment  (< 20% PPII by MD) | PPP Number of protein “hits”* | PGRS33  PPII helical  segment  (> 80% by MD) | PPP Number of protein “hits”* |
| --- | --- | --- | --- | --- | --- |
| GILIGN | 1 | PGTGAN | 2 | VGGAGG | 26 |
| AGLFGN | 1 | GAAGMP | 20 | NGGAGG | 23 |
| GLLYGA | 0 | GNVASG | 0 | AGGSGG | 24 |
| GLFFGV | 0 | GLAADA | 0 | DGGAGG | 26 |
| GLLFGA | 0 | TGTNVT | 0 | AGGNGG | 24 |
| GLFGVG | 2 | DGVAFL | 0 | DGGDGG | 31 |
| IGLVGN | 0 | DGVAFLGTAP | 2 | KGGDGG | 25 |
| GLAADA | 1 | SALLWG | 0 | GGAGGF | 25 |
| AGLLVGA | 0 | VGSTTG | 0 | GGAGGF | 29 |
| AGLLFG | 0 | AGALGGGATGV | 25 | NGGNGG | 23 |
| KAGLIG | 0 | RAGGGVGGI | 28 |  |  |
| GAILVG | 1 | GFGGAG | 21 |  |  |
| GLLGKN | 0 | NGTGAK | 0 |  |  |
|  |  | GSGTPN | 0 |  |  |

* The number of protein structures in the RCSB PDB with a six residue segment matching this sequence with an identity of at least 66%.

**Table S2.** Summary of CD results*

| **acPGPRGNQGAam** | | | | **acTRPEPNPVTam** | | | |
| --- | --- | --- | --- | --- | --- | --- | --- |
| Extra  Sample | [$\Theta]$ deg·cm^2^·dmol^-1^ at 220nm, 5.0ºC | %PPII | $\Delta$[$\Theta]$ deg·cm^2^·dmol^-1^  5.0ºC - 65ºC | Extra  Sample | [$\Theta]$ deg·cm^2^·dmol^-1^ at 224nm, 5.0ºC | %PPII | $\Delta$[$\Theta]$ deg·cm^2^·dmol^-1^  5.0ºC - 65ºC |
| 1 | -1639 | 32.6 | 1440 | 1 | -3861 | 16.3 | 2079 |
| 2 | -1837 | 31.1 | 1306 | 2 | -3946 | 15.7 | 2162 |
| 3 | -1835 | 31.1 | 1280 | 3 | -3710 | 17.4 | 2533 |
| 4 | -1948 | 30.3 | 1491 | 4 | -3884 | 16.2 | 2396 |
| **Mean** $\pm$**s.d.** | **-1815** $\pm$**129 (7.0%)** | **31.3%** $\pm$ **0.9%** | **1379** $\pm$ **102**  **(7.4%)** | **mean**$\pm$ **s.d.** | **-3850**$\pm$ **100 (2.6%)** | **16.4%** $\pm$ **0.7%** | **2293** $\pm$ **210**  **(9.2%)** |
| Values in Figure 6E | -1919 | 24% | 1831 | Values in Figure 6F | -4225 | 9% | 2684 |
| **Mean** $\pm$**s.d.**  **All values** | **-1815** $\pm$ **111**  **(6.1%)** | **29.8%** $\pm$ **3.4%** | **1470** $\pm$ **220**  **(15%)** | **Mean** $\pm$**s.d.**  **All values** | **-3925** $\pm$ **188**  **(4.8%)** | **14.9%** $\pm$ **3.3%** | **2371** $\pm$ **252**  **(10.6%)** |

*****Includes the results shown in **Figure 6** in the main text and four additional samples whose spectra are shown in **Figure S23**.

**Table S3**: Additional Benchmarking of PPP

| **Peptide/Protein Segment** | **NMR/CD**  **Xray diff.** | **AlphaFold** | **PPP Number of Matches Found^8^** | **Dynamine**  **% PPII** | **PPII Pred**  **% PPII** |
| --- | --- | --- | --- | --- | --- |
| PolyGly (G)_10_ | PPII helix^1^ | PPII, moderately high confidence^7^ | 26 | 26 | 8 |
| PolyPro (P)_10_ | PPII helix^2^ | PPII helix, high confidence^7^ | > 100 | 31 | 68 |
| PolyGlu (E)_10_ | PPII helix^2^ | some PPII helix, some coil^7^ | 2 | 10 | 11 |
| PolyLys (K)_10_ | PPII helix^2^ | some PPII helix, some coil^7^ | 8 | 9 | 10 |
| (GGM)_4_ | PPII helix, coil ^3^ | some PPII helix, some coil^7^ | 24 | 18 | 6 |
| Tau 1  T_175_PPAPKTPPSS_184_ | PPII helix ^4^ | coil, low confidence  AF-P10636-5-F1 | > 100 | 21 | 48 |
| Tau 2  P_216_TPPTREP_223_ | PPII helix ^4^ | coil, low confidence  AF-P10636-5-F1 | 81 | 19 | 25 |
| Tau 3  P_232_PKSPSSA_239_ | PPII helix ^4^ | coil, low confidence  AF-P10636-5-F1 | 71 | 18 | 20 |
| Measles Virus Nucleoprotein Ct1  G_411_PRQAQVSF_419_ | partial PPII helix ^5^ | coil / PPII helix, low confidence  AF-0000000365829749 | 8 | 11 | 6 |
| Measles V NP Ct2  S_505_EEQGSD_511_ | partial PPII helix ^5^ | α-helix / coil, low confidence  AF-0000000365829749 | 1 | 13 | 7 |
| CPEB3 1  P_86_PQQPPPPQ_94_ | PPII helix ^6^ | PPII helix, low confidence AF-Q8NE35-2-F1 | > 100 | 23 | 47 |
| CPEB3 2  P_164_PPPAPAPQP_175_ | PPII helix ^6^ | PPII helix, low confidence, AF-Q8NE35-2-F1 | > 100 | 23 | 56 |

^1^ The PPII structure of PolyGly was elucidated by Crick and Rich (1955) Nature **176**: 780-781.

^2^ The PPII nature of polyPro and of polyGlu and polyLys at neutral pH was established in the 1960s by the Tiffany and Krimm: Tiffany & Krimm (1968) Biopolymers **6**(9): 1379-1382; Tiffany & Krimm (1968) Biopolymers **6**(12): 1767-1770.

^3^ Based on papers identifying PPII helix in this sequence motif in Abductin: Bochicchio *et al.* (2005) Chirality **17**(7): 364-372 and GroEL: Rodríguez *et al.,* Protein Sci. 2025 **34**(11):e70354.

^4^ From Mukrasch *et al.,* (2009) PLoS Biol. **7**(2): e34.

^5^ From Ozenne *et al.,* (2012) J. Am. Chem. Soc. **134**(36): 15128-48

^6^ From Ramírez de Mingo *et al.,* (2022) BMC Biology **20**(1): 129

^7^ From inspection of the structural model generated by the AlphaFold server.

^8^ The number of protein structures in the RCSB PDB with a six residue segment matching this sequence with an identity of at least 66%.

**Table S4** NMR Spectral Parameters

| **Spectrum Type** | **FID Size** | **Number of Dummy / Recorded Scans** | **Mixing time (ms)** |
| --- | --- | --- | --- |
| 1D ^1^H | 32k | 4 / 32 | n.a. |
| 1D ^31^P | 2k | 4 / 32 | n.a. |
| 2D ^1^H-^1^H COSY | 8k x 512 | 48 / 8 | n.a. |
| 2D ^1^H-^1^H TOCSY | 2k x 512 | 48 / 8 | 60 |
| 2D ^1^H-^1^H NOESY | 2k x 512 | 32 / 48 | 80 |
| 2D ^1^H-^13^C HSQC* | 1k x 256 | 32 / 32 | n.a. |

n.a.: not applicable. * Recorded in 99.9% atom D D_2_O; other spectra were recorded in 90% H_2_O/10% D_2_O.
